# Lipidomic Profiling Reveals Distinct Molecular Signatures Across Clinical Subtypes of Myasthenia Gravis

**DOI:** 10.3390/metabo16080589

**Published:** 2026-08-19

**Authors:** Yufei Song, Die Dai, Min Cao, Rongrong Li, Jiaru Liu, Min Zhang, Yuqing Chen, Ruimin Tian, Peiyu Liu, Xiaoting Peng, Jiayi Huang, Qilin Fang, Beibei Dong, Biyi Pang, Liang Liu

**Affiliations:** 1 State Key Laboratory of Traditional Chinese Medicine Syndrome, The Second Affiliated Hospital of Guangzhou University of Chinese Medicine, The Second Clinical Medical School, Guangzhou University of Chinese Medicine, Guangzhou 510006, China; 20231110699@stu.gzucm.edu.cn (Y.S.); rongrongli@gzucm.edu.cn (R.L.); 20231110705@stu.gzucm.edu.cn (J.L.); 20231110704@stu.gzucm.edu.cn (M.Z.); 20231110700@stu.gzucm.edu.cn (Y.C.); tianruimin@gzucm.edu.cn (R.T.); 20232110197@stu.gzucm.edu.cn (P.L.); 20231110692@stu.gzucm.edu.cn (X.P.); 20231110696@stu.gzucm.edu.cn (J.H.); 20231110701@stu.gzucm.edu.cn (Q.F.); 20231110714@stu.gzucm.edu.cn (B.D.); 20231110698@stu.gzucm.edu.cn (B.P.); 2 Chinese Medicine Guangdong Laboratory (Hengqin Laboratory), Hengqin 519031, China; daidie@hqcmlab.cn; 3 School of Basic Medical Sciences, Guangzhou University of Chinese Medicine, Guangzhou 510006, China; caomin612@gzucm.edu.cn

**Keywords:** myasthenia gravis, diagnostic challenge, lipidomic signature, biomarkers, machine learning

## Abstract

Background/Objectives: Myasthenia gravis (MG) is an immune-mediated neuromuscular disorder for which antibody-based assays have limited sensitivity, particularly in double-seronegative MG (dsNMG), highlighting the need for complementary biomarkers. Given their roles in immune regulation, membrane integrity, and metabolic stress responses, lipids represent promising candidates for biomarker discovery. Methods: We designed a prospective case–control study and systematically stratified 68 patients with myasthenia gravis (MG) according to clinical classification and autoantibody status. Using LC–MS/MS, we quantified 824 lipids in 136 serum samples collected from these patients and 68 healthy controls. The analyzed subtypes included ocular MG (OMG), generalized MG (GMG), acetylcholine receptor antibody-positive MG (AChR-MG), and dsNMG. Differential lipid analysis, correlation network construction, KEGG pathway enrichment, and multivariable logistic regression were performed. Diagnostic and subtype prediction models were developed using LASSO with 10 × 10 repeated cross-validation and interpreted using Shapley Additive exPlanations (SHAP) analysis. A longitudinal follow-up analysis was conducted to assess dynamic associations between lipid signatures and disease activity. Results: In total, 240 lipids were significantly altered in MG compared with controls. Lipids distinguishing GMG from OMG were enriched in ether lipid metabolism, necroptosis, and sphingolipid signaling pathways. AChR-MG and dsNMG shared lipid networks related to membrane remodeling and signaling regulation, whereas dsNMG exhibited marked elevations in acylcarnitines and bile acid-related metabolites, potentially reflecting a distinct phenotype characterized by altered energy metabolism. The lipid-based model achieved an AUC of 0.917 for distinguishing MG from controls, and AUCs of 0.77 and 0.71 for differentiating AChR-MG from dsNMG and GMG from OMG, respectively. Longitudinal analyses showed that SM(d18:1/23:0) and Cer(d24:1/18:0(2OH)) displayed dynamic changes consistent with disease activity. Conclusions: Serum lipidomics revealed subtype-specific metabolic features of MG, with stable disease-associated remodeling and dynamic sphingolipid changes potentially reflecting disease activity. By integrating systematic clinical and antibody-based subtype stratification with longitudinal follow-up, this study supports lipidomics as a complementary tool for precision diagnosis and disease stratification, particularly in antibody-negative dsNMG.

## 1. Introduction

Myasthenia gravis (MG) is an autoimmune disorder caused by the production of pathogenic autoantibodies targeting proteins at the neuromuscular junction [1]. Clinical manifestations include ptosis, diplopia, and muscle weakness [2]. Acetylcholine receptor antibody (AChR-Ab) and muscle-specific kinase antibody (MuSK-Ab) are recognized diagnostic biomarkers [3]. Conventional diagnostic methods can detect AChR-Ab in about 70% of MG patients and MuSK-Ab in approximately 1–10% of patients. Yet, approximately 15% of generalized MG (GMG) patients are seronegative, even with a higher proportion among ocular MG (OMG) [4]. Moreover, antibody titers do not consistently correlate with disease severity [5]. Patients negative for both AChR-Ab and MuSK-Ab are classified as having double-seronegative myasthenia gravis (dsNMG) [6]. The absence of detectable biomarkers complicates the diagnosis and clinical management of dsNMG. Therefore, there is a need for identifying novel biomarkers to support diagnosis and guide treatment.

Given that myasthenia gravis is a rare disease, most existing studies are characterized by relatively small sample sizes, commonly comprising 20–60 participants [7,8,9], and they generally focus on overall differences between patients with MG and healthy controls, with insufficient exploration of clinical heterogeneity regarding the disease. However, few studies have systematically compared MG subtypes, particularly OMG and GMG, or examined antibody-defined subgroups. In particular, studies on dsNMG are scarce, leaving its metabolic characteristics, underlying mechanisms, and potential biomarkers largely unexplored. Therefore, conducting comprehensive subtype comparisons is essential for understanding the heterogeneity of MG and improving clinical diagnosis.

Lipids participate in diverse biological processes, including cell signaling, phenotypic regulation, and immune responses in disease contexts [10,11,12]. Evidence suggests that in autoimmune and inflammatory diseases, lipids could influence disease progression by modulating energy metabolism, altering membrane structure and function, and regulating immune activity [13,14,15]. Previous studies have reported marked metabolic differences between MG patients and healthy individuals, as well as between pre- and post-treatment states [16,17,18], suggesting that dysregulated lipid metabolism may contribute to MG onset and progression. Previous studies identified systemic metabolic alterations in MG but rarely addressed disease heterogeneity [16,17,18]. Lipid changes across clinical and antibody-defined subtypes, particularly dsNMG, and their longitudinal relationships with disease activity remain unclear. The key strength of this study is its deeply phenotyped cohort, integrating subtype stratification with longitudinal follow-up to evaluate lipid alterations across MG phenotypes and clinical states.

To address these gaps, we performed comprehensive lipidomic profiling and integrated clinical and antibody-based stratification. This study provides a comprehensive characterization of lipid alterations across clinical and antibody-defined MG subtypes, complemented by longitudinal follow-up. This strategy enabled the identification of dysregulated pathways and molecular patterns associated with disease heterogeneity. On this basis, longitudinal follow-up analyses were further incorporated to evaluate the dynamic associations between MG-associated lipid signatures identified in the cross-sectional analyses and clinical improvement over the course of disease. Furthermore, we developed and validated a lipid-based diagnostic prediction model to evaluate the potential of lipidomic features as biomarkers for distinguishing MG and its subtypes, with particular emphasis on differentiating double-seronegative MG (dsNMG) from healthy controls. This integrated framework may identify complementary biomarkers for antibody-negative MG and support precision diagnosis, subtype stratification, and disease monitoring.

## 2. Materials and Methods

### 2.1. Patient Recruitment

This was a prospectively conducted, non-interventional observational case–control study. From April 2024 to April 2025, a total of 68 patients with MG and 68 healthy controls were recruited at the University Town Hospital of Guangdong Provincial Hospital of Traditional Chinese Medicine. Patients with MG were diagnosed according to internationally accepted criteria. The diagnosis of MG was established based on characteristic clinical features and the presence of one or more of the following findings: (1) positive serum AChR or MuSK antibodies; (2) electrophysiological evidence of impaired neuromuscular transmission, such as decremental responses on repetitive nerve stimulation or single-fiber electromyography abnormalities; and (3) clinical improvement following cholinesterase inhibitor administration. Patients were excluded if they had comorbid autoimmune diseases, active infections, or had received antibody-removing therapies such as plasma exchange or intravenous immunoglobulin within three months before enrollment. Age- and sex-matched healthy controls were recruited as a comparison group. Within the MG cohort, 27 patients completed a 6-month longitudinal follow-up with paired serum samples obtained at baseline and at the follow-up visit. Patients were subsequently categorized based on changes in Myasthenia Gravis Activities of Daily Living (MG-ADL) scores over the follow-up period. Clinical improvement was defined as a reduction in MG-ADL score of ≥2 points at 6 months (ΔADL6 ≥ 2), while patients not meeting this criterion were classified as not improved [19]. The study was conducted in accordance with the Declaration of Helsinki and approved by the Ethics Committee of Guangdong Provincial Hospital of Chinese Medicine (Approval Code: YF2024-140-01; Approval Date: 31 May 2024). Written informed consent was obtained from all participants.

### 2.2. Clinical Assessment

At enrollment, each patient with MG underwent comprehensive clinical evaluation, including: (1) documentation of clinical features and classification using the Myasthenia Gravis Foundation of America (MGFA) system; (2) recording of disease duration and treatment status, including use of immunosuppressants or cholinesterase inhibitors; (3) assessment of thymic or thyroid abnormalities and thymectomy history; (4) assessment of the MG-ADL score by a trained clinician; and (5) collection of 10 mL of fasting venous blood. Clinical subtypes were classified according to the Myasthenia Gravis Foundation of America (MGFA) clinical classification. Ocular MG (OMG) was defined as weakness limited to the ocular muscles, corresponding to MGFA class I, whereas generalized MG (GMG) was defined as involvement of bulbar, limb, axial, or respiratory muscles in addition to or irrespective of ocular manifestations, corresponding to MGFA classes II–V. Antibody-defined subtypes were determined according to serum AChR-Ab and MuSK-Ab status. AChR-MG and MuSK-MG were defined by positivity for the corresponding antibody, whereas double-seronegative MG (dsNMG) was defined by negative results for both AChR-Ab and MuSK-Ab.

### 2.3. Serum Collection and Autoantibody Assay

Fasting peripheral venous blood samples were collected from all MG patients. After clotting at room temperature for 30–45 min, samples were centrifuged at 3500 rpm for 10 min at 4 °C. Serum was aliquoted into 0.6 mL Eppendorf tubes (≤200 µL per tube) and stored at −80 °C until analysis. Acetylcholine receptor antibody (AChR-Ab) and muscle-specific kinase antibody (MuSK-Ab) levels were measured for all MG patients. AChR-Ab concentrations were determined using a competitive inhibition ELISA kit (RSR Ltd., Cardiff, UK; Cat. #KAE113). MuSK-Ab levels were assessed with a competitive binding ELISA kit (Tecan, Hamburg, Germany; Cat. #RE51021). All assays were performed in duplicate according to manufacturer’s instructions, and mean values were used for analysis.

### 2.4. Serum Lipidomic Analysis

Serum lipids were extracted using a modified methyl tert-butyl ether (MTBE)-based protocol established by Wuhan Metware Biotechnology Co., Ltd., Wuhan, China, based on the method described by Matyash et al. [20]. Serum samples were thawed on ice and vortexed for 10 s. A 50-μL serum aliquot was mixed with 1 mL of extraction solvent consisting of MTBE and methanol (3:1, *v*/*v*) and containing an internal standard mixture. The resulting solvent-to-sample ratio was 20:1 (*v*/*v*). The identities, lipid classes, working concentrations, and amounts of the internal standards added to each sample are provided in Supplementary Table S1. Samples were vortexed for 15 min. Subsequently, 200 μL of ultrapure water was added to induce phase separation. The samples were vortexed for an additional 1 min and centrifuged at 12,000 rpm for 10 min. A 200-μL aliquot of the upper organic phase was collected and evaporated to dryness using a vacuum concentrator. The dried extract was reconstituted in 200 μL of acetonitrile/isopropanol (1:1, *v*/*v*) before UPLC–MS/MS analysis.

Chromatographic separation was performed using an ExionLC™ AD UPLC system coupled to a QTRAP^®^ 6500+ mass spectrometer (SCIEX, Framingham, MA, USA). Lipids were separated on a Thermo Accucore™ C30 column (2.1 × 100 mm, 2.6 μm; Thermo Fisher Scientific, Waltham, MA, USA) maintained at 45 °C. Mobile phase A consisted of acetonitrile/water (60:40, *v*/*v*) containing 0.1% formic acid and 10 mmol/L ammonium formate, whereas mobile phase B consisted of acetonitrile/isopropanol (10:90, *v*/*v*) containing 0.1% formic acid and 10 mmol/L ammonium formate. The gradient program, expressed as the volume ratio of mobile phases A/B, was as follows: 80:20 at 0 min, 70:30 at 2 min, 40:60 at 4 min, 15:85 at 9 min, 10:90 at 14 min, 5:95 at 15.5–17.3 min, and 80:20 at 17.5–20 min. The flow rate was 0.35 mL/min, and the injection volume was 2 μL.

Data were acquired in multiple reaction monitoring (MRM) mode under positive and negative electrospray ionization conditions. The ion-source temperature was 500 °C, and the ion-spray voltages were +5500 and −4500 V in the positive- and negative-ion modes, respectively. The curtain gas, ion source gas 1, ion source gas 2, and collision gas were set to 35, 45, 55, and 35 psi, respectively.

The MRM method was constructed using the in-house Metware Database (MWDB), which contains compound-specific Q1–Q3–RT–DP–CE parameters. MRM transitions were selected according to established lipid fragmentation patterns and MS-DIAL resources, and DP and CE were optimized to improve the signal-to-noise ratios of characteristic product ions. Retention times were determined using high-resolution MS/MS spectra and available authentic standards, with 54 lipid standards used for retention-time calibration. For lipids without authentic standards, retention times were predicted from the elution patterns of structurally related lipids. The MRM detection window was set to 45 s, and lipid annotation required agreement with the characteristic Q1–Q3 transition and a retention-time deviation within ±0.2 min. MWDB was used to construct the targeted MRM method and support lipid annotation, rather than for untargeted database searching. Lipids were reported using LIPID MAPS nomenclature [21].

Lipid abundances were reported on a relative, semi-quantitative basis rather than as absolute concentrations. Lipid abundance was calculated as *X* = 0.001 × *R* × *c* × *F* × *V*/*m*, where *X* is the estimated lipid concentration (nmol/mL), *R* is the analyte-to-internal-standard peak-area ratio, *c* is the internal-standard concentration, *F* is the lipid-class-specific correction factor, *V* is the extraction volume, and *m* is the serum volume.

Pooled quality-control (QC) samples were prepared by mixing equal aliquots of all samples within each analytical run. One QC sample was injected after every ten study samples, yielding a total of 21 QC injections. The pooled QC samples were used solely to monitor analytical stability and calculate feature-level coefficients of variation (CVs), rather than for sample normalization. Lipid features with CVs <30% in the QC samples were retained for subsequent statistical analyses. All baseline and longitudinal samples were analyzed using the same UPLC–MS/MS platform, analytical conditions, and QC procedures.

### 2.5. Differential Lipid Analysis

We performed multivariate and univariate analyses on normalized lipidomic data to identify differential lipids between MG subgroups and HCs. The two patients with MuSK-MG were retained in analyses of the overall MG cohort and in MGFA-based comparisons according to their clinical classification. However, because the MuSK-MG subgroup was too small for reliable subgroup-level inference (*n* = 2), these patients were excluded from antibody-defined comparative analyses. They were not included in the dsNMG group, which was restricted to patients negative for both AChR-Ab and MuSK-Ab.

Lipid abundances were log10-transformed and unit variance-scaled to minimize technical bias. PCA was performed using the prcomp function in R (v4.4.0) to visualize overall distributions. Overall differences in multivariate lipidomic profiles were evaluated separately for the HCs–OMG–GMG and HCs–AChR-MG–dsNMG classifications using PERMANOVA with Euclidean distances and 999 permutations (vegan package, v2.6.4). Differences in PC1 and PC2 scores were assessed using Welch’s one-way ANOVA. When the overall test was significant, pairwise comparisons were performed using the Games–Howell post hoc test. For univariate analysis, pairwise comparisons between MG subtypes and HCs (or between subtypes) were performed using the Wilcoxon rank-sum test. OPLS-DA models were constructed for each pairwise comparison, and model performance was assessed using R^2^X(cum), R^2^Y(cum), and Q^2^(cum). To evaluate potential overfitting, 200 class-label permutations were performed for each model. Empirical permutation *p* values were calculated as the proportion of permuted models with R^2^Y or Q^2^ values equal to or greater than the corresponding value of the original model. Models with a positive Q^2^ value and significant permutation tests for both R^2^Y and Q^2^ were considered to show adequate predictive validity. OPLS-DA-derived VIP values were used for feature prioritization only when the corresponding model passed permutation-based validation. Lipids were considered significantly different if variable importance in projection (VIP) >1 from OPLS-DA and the Wilcoxon test *p* < 0.05, with regulation direction determined by group mean differences. Visualization included PCA score plots and volcano plots using ggplot2 (v3.5.0), ggpubr (v0.6.0), and patchwork.

A hierarchical clustered heatmap was generated using ComplexHeatmap based on the normalized lipid matrix. Missing values were imputed with row medians. For three pairwise comparisons (MG vs. HCs, OMG vs. GMG, dsNMG vs. AChR-MG), Cohen’s d effect sizes were computed per lipid, and the maximum |d| was retained. Lipids with |d| ≥ 0.40 or in the top 40% of effect sizes were selected and row Z-score-standardized, and low-variance lipids (bottom 30% post-Z variance) were excluded. Column annotations included Group, MGFA classification, and antibody subtype, with binary clinical variables encoded as Yes/No.

For visualization, the five top-ranked non-duplicated lipids from each subtype comparison (GMG vs. OMG and AChR-MG vs. dsNMG) were selected. Overall differences were assessed separately for HCs–OMG–GMG and HCs–AChR-MG–dsNMG using Welch’s ANOVA, followed by Games–Howell pairwise tests with Benjamini–Hochberg FDR correction across the ten lipids.

### 2.6. Lipid Correlation Network Analysis

We constructed correlation-based lipid interaction networks to explore coordinated lipid changes among MG subtypes and HCs. Lipid abundances were log_2_-transformed and row Z-score normalized. Spearman correlations were calculated for all lipid pairs within each group, with Benjamini–Hochberg correction. Significant correlations (|ρ| ≥ 0.60, FDR ≤ 0.05) were retained to build networks, where nodes represented differential lipids and edges represented correlations. Node degree, betweenness centrality, and Louvain community detection were used for topological analysis. Networks were visualized in Gephi software (version 0.10.1).

### 2.7. KEGG Pathway Enrichment Analysis of Differential Lipids

KEGG pathway enrichment analysis was performed on differential lipids to identify functional alterations in lipid metabolism across MG subtypes. For MGFA subtype analysis, the union of differential lipids from HCs vs. OMG, HCs vs. GMG, and OMG vs. GMG was used. For antibody subtype analysis, the union from HCs vs. AChR-MG, HCs vs. dsNMG, and AChR-MG vs. dsNMG was used. Enrichment was assessed via hypergeometric tests based on the KEGG database. Pathways were ranked by *p*-values, and significance was visualized as −log_10_(*p*). Analyses were conducted on the Metware Cloud platform (https://cloud.metware.cn, accessed on 12 August 2026).

### 2.8. Lipid Association Analysis

We evaluated associations between lipids and MG clinical phenotypes using multivariable logistic regression for three comparisons: MG vs. HCs, OMG vs. GMG, and AChR-MG vs. dsNMG. Lipid intensities were log-transformed and Z-score normalized, then integrated with clinical covariates (age, sex, thymic disease, thyroid dysfunction, and immunosuppressive treatment). For each comparison, a logistic regression model was fitted with disease status as the dependent variable. Odds ratios (OR) and Wald 95% CIs were calculated for each lipid. Significance was defined as *p* < 0.05. Significant lipids were visualized using forest plots and mapped to KEGG pathways for functional interpretation.

### 2.9. Diagnostic Model Construction and Evaluation

We built binary diagnostic models using lipidomic features to distinguish MG and its subtypes from HCs. Analyses were performed in R using glmnet, caret, pROC, and fastshap. Lipid data were median-imputed, reshaped into a sample × feature matrix, and column-standardized. LASSO logistic regression with 10-fold cross-validation was applied to the entire study cohort for feature selection before repeated cross-validation (lambda.1se or lambda.min if no features were selected). Thus, feature selection was not independently repeated within the training subset of each cross-validation iteration. The top three features were used for modeling; if fewer were available, additional lipids were included based on their highest absolute correlation with class labels or variance. Separate models were developed for each comparison. Multivariable logistic regression classifiers were evaluated via repeated stratified K-fold cross-validation (K = min [10, minority-class size]), repeated 10 times. Standardization parameters were recalculated within each training fold. Performance was assessed using ROC-AUC, with overall AUC and 95% DeLong CIs reported. Fold-level AUC distributions summarized model stability, and combined ROC curves enabled cross-model comparison.

SHAP analyses were performed to quantify the contribution of individual lipid features to model classification. For each binary classification model, SHAP values were calculated on the predicted-probability scale for the predefined positive class. Positive SHAP values indicate that a lipid feature shifts the model output toward the positive class, whereas negative values shift the output toward the reference class. The point color in the SHAP summary plots represents the standardized abundance of the corresponding lipid feature.

## 3. Results

### 3.1. Construction of the Experimental and Analytical Workflow

**Figure 1:**
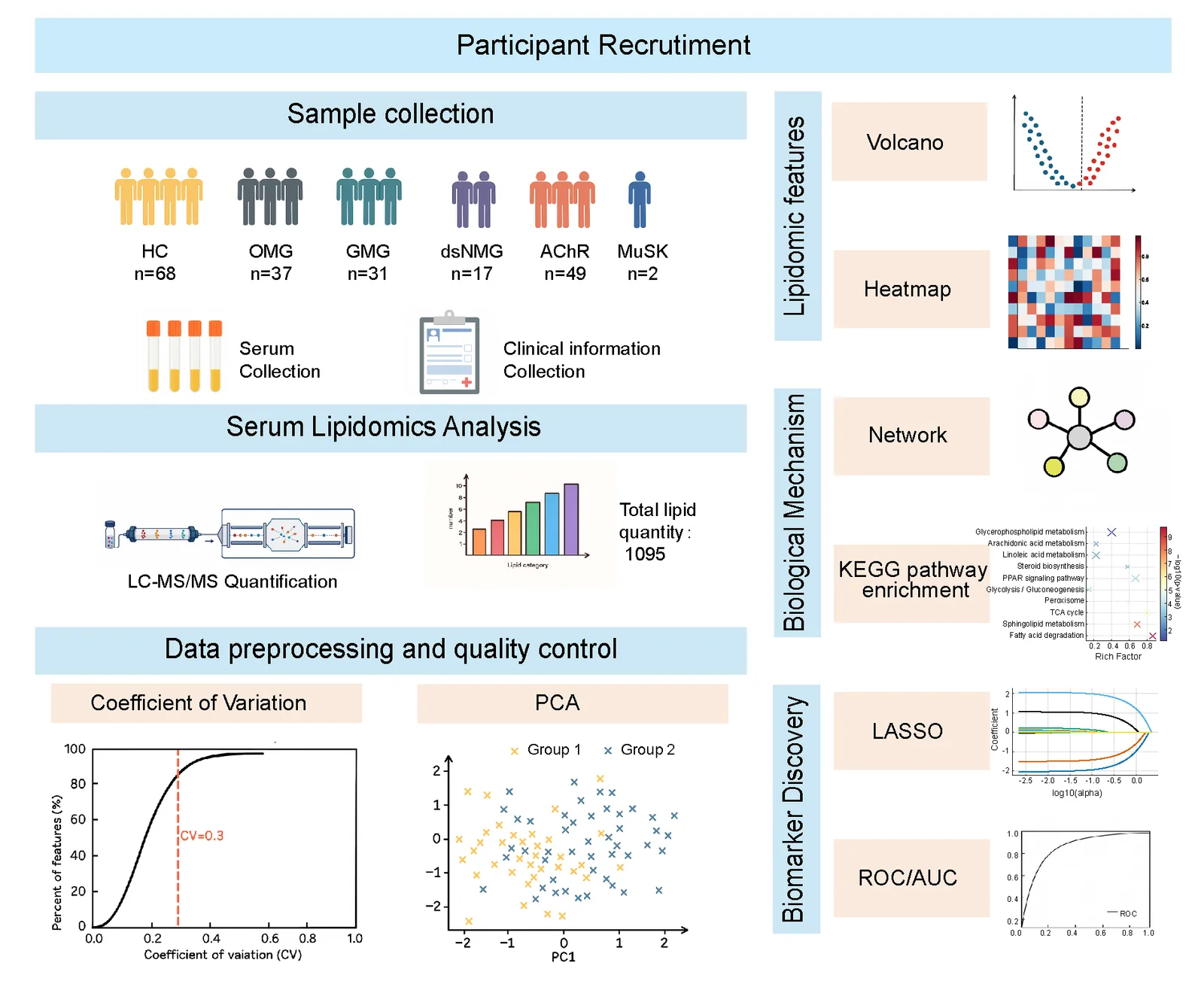
Study workflow. The schematic diagram outlines the key experimental and analytical procedures. Briefly, 68 patients with myasthenia gravis (MG) and 68 healthy controls (HCs) were recruited. MG patients were further stratified into clinical subtypes (ocular MG [OMG, *n* = 37] and generalized MG [GMG, *n* = 31]) and antibody-based subtypes (acetylcholine receptor antibody-positive MG [AChR-MG, *n* = 49], muscle-specific kinase-positive [MuSK-MG, *n* = 2], and double-seronegative MG [dsNMG, *n* = 17]). Serum samples from all participants underwent lipidomic profiling using LC-MS/MS, which identified 1095 lipid metabolites. After stringent quality control (coefficient of variation < 30%), 824 lipids were retained for downstream analysis. These analyses included exploration of lipidomic profiles, investigation of biological mechanisms via lipid correlation network and KEGG pathway analyses, and biomarker discovery and model validation using LASSO regression and receiver operating characteristic (ROC) curves. Elements of this figure were generated with the assistance of artificial intelligence and were subsequently reviewed and edited by the authors to ensure scientific accuracy.

**Figure 2:**
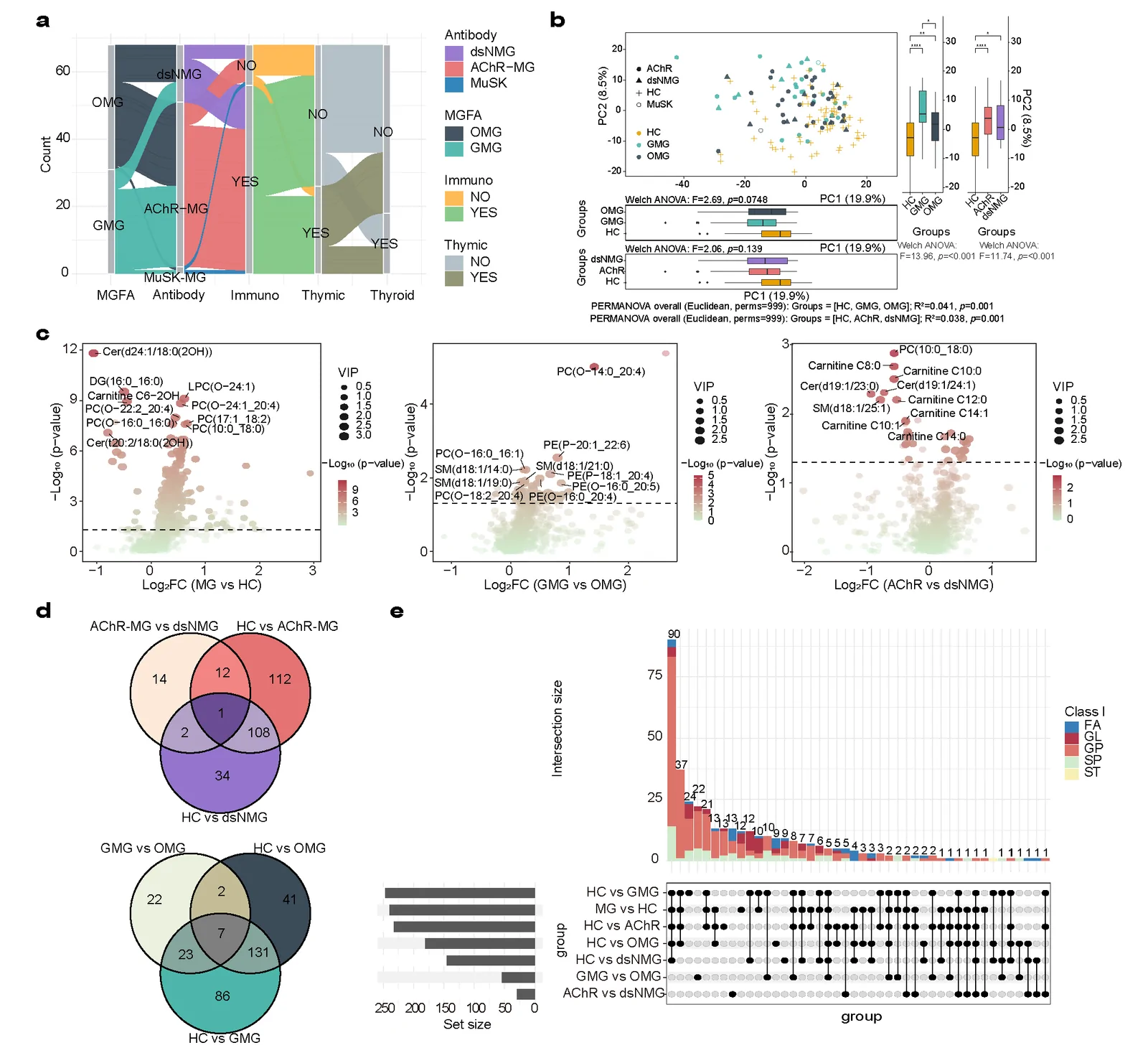
Lipidomic profile of MG patients and subtypes. (**a**) Alluvial plot showing the distribution and transitions of clinical phenotypes among MG patients (*n* = 68). Each colored band represents a distinct clinical feature, and band width corresponds to the number of patients. (**b**) Integrated PCA of serum lipidomic profiles. The central plot shows individual samples, with colors indicating MGFA groups and symbols indicating antibody subtypes. Marginal boxplots compare PC1 and PC2 scores across MGFA- and antibody-defined groups. Overall separation was assessed by PERMANOVA, followed by Welch’s ANOVA and Games–Howell tests for PC scores. * *p* < 0.05; ** *p* < 0.01; **** *p* < 0.0001. (**c**) Volcano plots show differentially abundant lipids across different comparison groups. Significant lipids were defined as *p* < 0.05 (Wilcoxon test) and VIP > 1 (OPLS-DA); the top ten lipids are annotated. The horizontal dashed line indicates the significance threshold. (**d**) Venn diagrams showing shared and unique differentially abundant lipids across subgroup comparisons, with values indicating the number of lipids in each overlap. (**e**) UpSet plot summarizing seven pairwise intersections of differential lipids. Vertical bars represent intersection sizes and are stratified by lipid class (Class I). Bar charts on the left indicate the total number of differential lipids per comparison.

The overall experimental and analytical workflow is summarized in Figure 1. A total of 136 participants were enrolled in this study, comprising 68 MG patients and 68 HCs. Patients were stratified along two dimensions: clinically, according to the Myasthenia Gravis Foundation of America (MGFA) classification, into OMG (*n* = 37) or GMG (*n* = 31); immunologically, based on antibody status, into AChR-MG (*n* = 49), MuSK-MG (*n* = 2), or dsNMG (*n* = 17) (Table 1). Owing to the limited number of MuSK-MG patients, they were retained in the overall MG and MGFA-based analyses but were not analyzed as an independent antibody-defined subgroup or included in comparisons between AChR-MG and dsNMG. Figure 2a illustrates the distribution of MG patients across five variables: MGFA classification, antibody subtype, immunosuppressive therapy, thymic abnormalities, and thyroid disease. It can be observed that most GMG patients and those with thymic abnormalities are concentrated in the AChR-MG subgroup, whereas OMG patients are more frequently distributed within the dsNMG subgroup. The overall distribution pattern aligns with the general epidemiological characteristics of MG [6], indicating that this cohort possesses good clinical representativeness. HCs were matched to the patient group by age and sex. Among these patients, 27 individuals with MG were further included in a longitudinal follow-up analysis to provide internal temporal validation of the associations between lipidomic features identified in the cross-sectional analyses and disease activity.

Serum lipids were detected using ultra-performance liquid chromatography-tandem mass spectrometry (UPLC-MS/MS), and differential lipid analysis was subsequently performed. To explore commonly and uniquely dysregulated biological pathways and key lipids across MG subtypes, lipid correlation networks and Kyoto Encyclopedia of Genes and Genomes (KEGG) pathway analyses were conducted. Additionally, logistic regression analysis was performed to identify disease-associated lipids, comparing MG versus HCs as well as across different subtypes. The models were adjusted for age, sex, thymic disease, thyroid disease, and immunosuppressive treatment, with a *p* < 0.05 considered statistically significant. Finally, following feature selection via the least absolute shrinkage and selection operator (LASSO), logistic regression models were constructed to evaluate the diagnostic potential of lipid signatures for MG and its subtypes. Furthermore, a longitudinal follow-up analysis was conducted to evaluate the dynamic associations between MG-associated lipid signatures and disease activity.

In summary, this study employed a cross-sectional lipidomics approach combined with longitudinal follow-up validation to systematically characterize the lipid metabolic profiles of MG and its subtypes, including assessments of disease status, clinical severity, and immunophenotype.

### 3.2. Differential Lipidomic Profiles in the Subtypes of Myasthenia Gravis and Healthy Controls

A total of 824 of the 1095 detected lipid features passed quality-control filtering and were retained for downstream analyses. These lipids spanned six major categories: glycerolipids, glycerophospholipids, sphingolipids, prenol lipids, sterol esters, and fatty acyls. Among them, glycerophospholipids (48.2%), glycerolipids (30.4%), and sphingolipids (13.5%) constituted the dominant classes (Supplementary Figure S1a), consistent with the broad representation of these categories in comprehensive analyses of the human plasma lipidome [22]. The six major lipid classes were further resolved into 43 lipid subclasses, whose proportional compositions are shown in Supplementary Figure S1b.

We first assessed global differences in lipidomic profiles among MG subgroups and HCs. The PCA showed partial separation among the study groups (Figure 2b). PERMANOVA detected statistically significant differences among HCs, GMG, and OMG (R^2^ = 0.041, *p* = 0.001), as well as among HCs, AChR-MG, and dsNMG (R^2^ = 0.038, *p* = 0.001). However, the low R^2^ values indicate that, although the between-group differences were statistically significant, group membership explained only a small proportion of the overall interindividual variation. The accompanying marginal boxplots showed the distributions of PC1 and PC2 scores across the corresponding subgroups, with significant pairwise differences evaluated using Games–Howell post-hoc tests, indicating modest associations between the principal axes of lipidomic variation and the clinical or antibody-defined classifications. The OPLS-DA models comparing MG or individual MG subtypes with healthy controls (HCs) showed positive Q^2^ values (0.455–0.622), with significant permutation tests for both R^2^Y and Q^2^. In contrast, the OMG versus GMG and AChR-MG versus dsNMG models yielded negative Q^2^ values (−0.212 and −0.280, respectively) and nonsignificant permutation tests, indicating limited predictive performance. Complete validation results are provided in Supplementary Table S2.

Differential analysis identified distinct metabolic signatures between each MG subgroup and HCs. In total, 240 differential lipids were associated with MG overall (223 upregulated, 17 downregulated), 54 lipids differed by MGFA classification (53 upregulated, 1 downregulated), and 29 lipids differed by antibody subtype (12 upregulated, 17 downregulated). Specifically, compared with HCs, 233 differential lipids were detected in AChR-MG (23 upregulated, 210 downregulated), 145 in dsNMG (11 upregulated, 134 downregulated), 181 in OMG (16 upregulated, 165 downregulated), and 247 in GMG (13 upregulated, 234 downregulated). Additional comparisons are presented in Supplementary Figure S2. The specific list of differential lipids is provided in Supplementary Table S3. These findings indicate a profound restructuring of lipid metabolism in MG, consistent with previous metabolomic and clinical lipid studies [17,18].

A categorical analysis of differential lipids across each comparison group revealed that differential lipid profiles were predominantly driven by GP (44–77%), followed by SP (12–28%), whereas fatty acyls (FA) and GL, despite their relatively lower abundance (6–34%), exhibited substantial disease-associated variation (Supplementary Figure S3). Among these alterations, ceramide dysregulation was particularly prominent. Consistent with our findings, a previous MG-specific study demonstrated that several plasma ceramides were elevated in patients with MG, correlated with QMG and MG-ADL scores, and decreased in parallel with clinical improvement [23]. Comparison of GMG vs. OMG revealed accumulation of ether-linked PC(O-) and PE(P-), indicative of increased membrane remodeling and oxidative stress, in agreement with the proposed antioxidant and membrane-protective properties of plasmalogens [24]. In contrast, differential metabolites associated with antibody subtype included elevations in carnitines such as C8:0 and C10:0, a pattern consistent with altered mitochondrial fatty acid metabolism [25].

To delineate shared and subtype-specific metabolic alterations, we constructed Venn and UpSet diagrams based on differential lipid sets (Figure 2d,e; Supplementary Table S4). A total of 108 lipids were commonly altered in both AChR-MG and dsNMG compared with HCs, forming a core disease-related lipid pool dominated by phospholipids, ceramides, and sphingomyelins—suggesting a potential involvement of membrane remodeling and sphingolipid-mediated signaling alterations shared across antibody subtypes [26]. AChR-MG exhibited 112 unique differential lipids primarily from phospholipid subclasses, whereas dsNMG showed distinctive alterations in triglycerides (TG) and sphingomyelin subclasses, along with the emergence of glycocholic acid, suggesting a distinct lipid profile in dsNMG. Among 22 lipids distinguishing OMG from GMG, ether-linked PE(O-)/PE(P-) and multiple phosphatidic acid (PA) species were prominent, supporting an association between GMG severity and lipid patterns related to oxidative stress and inflammatory processes [24]. GMG-specific changes (*n* = 86) further involved synaptic membrane sphingolipids and multi-layer phospholipid remodeling, suggesting a potential association with broader neuromuscular junction involvement and systemic clinical phenotypes.

Finally, effect-size filtering and variance stabilization enabled hierarchical clustering (Supplementary Figure S4), revealing distinct stratification not only between MG and controls, but also among MG subtypes. Differential lipids were predominantly distributed across the GP, SP, and FA subclasses. This pattern reflects the subclass distribution of differential features rather than subclass-wide increases in abundance, because most individual differential lipids were decreased relative to HCs.

### 3.3. Functional Correlations and Mechanistic Implications of Lipid Dysregulation in MG

**Figure 3:**
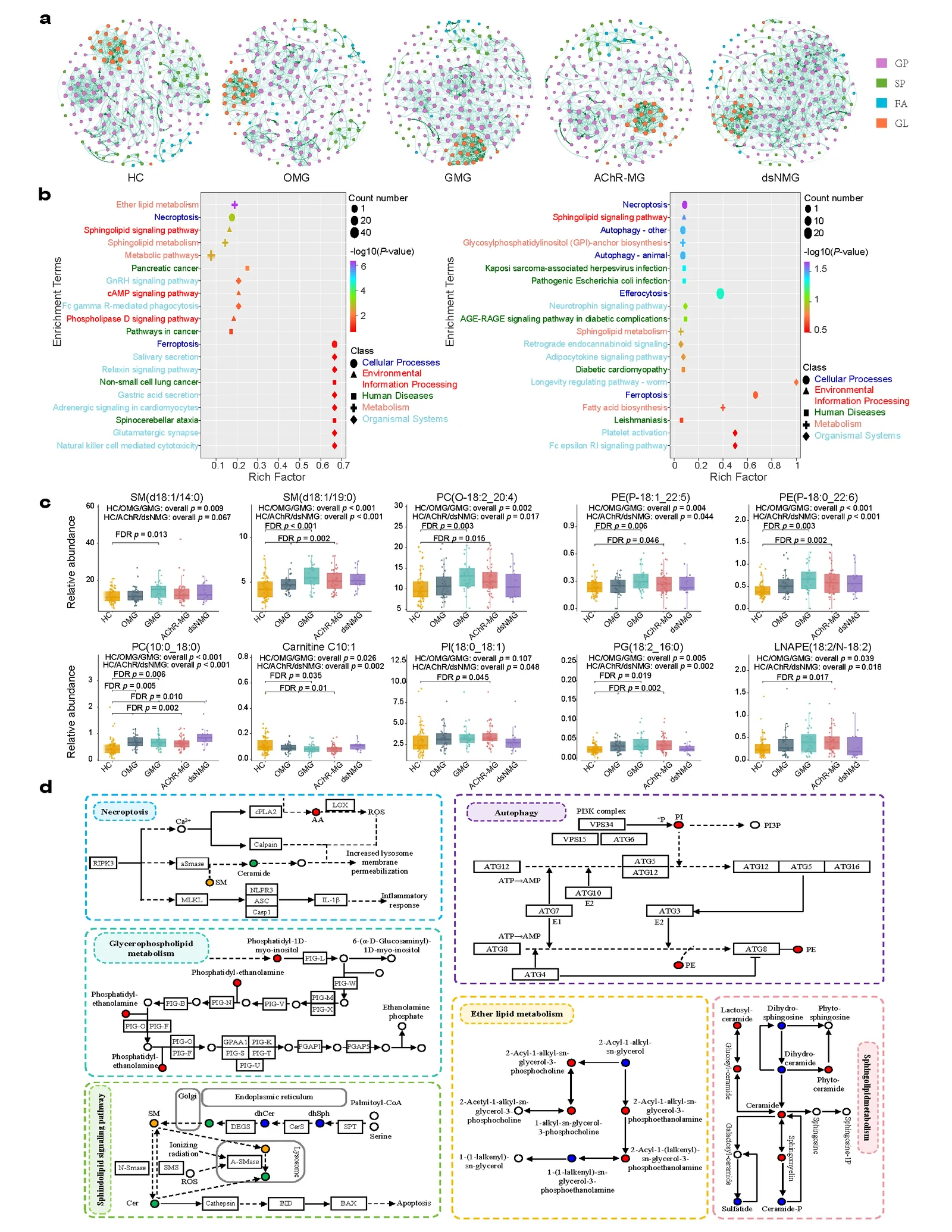
Functional enrichment and mechanistic implications of lipid dysregulation in MG. (**a**) Correlation network illustrating coordinated lipid remodeling. Differential lipids were evaluated using Spearman correlations, retaining edges with |ρ| ≥ 0.60 and Benjamini–Hochberg FDR ≤ 0.05. Node color denotes lipid class, node size reflects degree centrality, and edge width corresponds to correlation strength. GP: glycerophospholipids; SP: sphingolipids; FA: fatty acyls; GL: glycerolipids. (**b**) KEGG pathway enrichment. (**Left**): pathways associated with MGFA subtypes (HCs vs. OMG, HCs vs. GMG, OMG vs. GMG). (**Right**): pathways associated with antibody subtypes (HCs vs. AChR-MG, HCs vs. dsNMG, AChR-MG vs. dsNMG). Bubble size represents the number of enriched lipids, color indicates statistical significance, and shape denotes pathway category. (**c**) Distribution of ten representative lipids across myasthenia gravis subgroups. Overall differences were assessed separately among HCs, OMG, and GMG and among HCs, AChR-MG, and dsNMG using Welch’s ANOVA. *p* values from Games–Howell pairwise comparisons across the ten lipids were adjusted using the Benjamini–Hochberg method, and results with FDR < 0.05 are indicated. (**d**) Mechanistic schematic linking lipid imbalance to MG pathology. The diagram maps altered lipids to relevant pathways; red denotes upregulation, blue downregulation, green/orange highlight critical enzymes or intermediates, and white indicates no change or undetected molecules.

We first conducted a systematic evaluation of the expression associations of differential lipids across subgroups (HCs, OMG, GMG, AChR-MG, and dsNMG). Lipid-lipid correlation networks (Figure 3a) revealed striking subtype-specific remodeling of lipid interactions. In HCs, GP formed tightly clustered communities, whereas in MG patients these networks became more dispersed, indicating a loss of coordinated phospholipid regulation. Conversely, GL exhibited denser clustering in MG than in HCs, suggesting a disease-specific reorganization of storage and membrane-associated lipid metabolism. Together, these patterns highlight substantial lipid network reconfiguration underlying MG.

We next compared pathway enrichment profiles of differential lipids across MG subtypes. Both dimensions of stratification demonstrated shared and distinct metabolic features. Lipids associated with MGFA clinical subtypes (Figure 3b Left) were primarily enriched in Necroptosis, Sphingolipid signaling pathway, Autophagy, and Glycosylphosphatidylinositol (GPI)-anchor biosynthesis. In contrast, antibody-based subgroup differences (Figure 3b Right) were enriched in Ether lipid metabolism, Necroptosis, Sphingolipid signaling pathway, and Sphingolipid metabolism. Notably, Necroptosis and sphingolipid signaling were shared across both stratification dimensions, indicating that pathways related to programmed cell death and immune signaling are consistently associated with MG subtype differences [23,27].

Boxplots of the ten representative lipid features (Figure 3c) further illustrated differences across the two clinical classification systems. Overall differences were evaluated separately for the HCs–OMG–GMG and HCs–AChR-MG–dsNMG groupings using Welch’s one-way ANOVA. Significant pairwise differences were subsequently examined using Games–Howell tests with Benjamini–Hochberg FDR correction across the ten lipid features. Several sphingomyelin and glycerophospholipid species, including SM(d18:1/19:0), PC(O-18:2_20:4), PE(P-18:1_22:5), PE(P-18:0_22:6), PC(10:0_18:0), PG(18:2_16:0), and LNAPE(18:2/N-18:2), showed significant differences in at least one classification system. Carnitine C10:1 and PI(18:0_18:1) also exhibited classification-dependent variation. These results indicate that the selected lipid features capture both MGFA-related and antibody-related metabolic heterogeneity.

To further elucidate the biological implications of these lipid alterations, significantly dysregulated species were mapped to functional lipid modules (Figure 3d). Overall, the results indicate that lipid metabolic reprogramming in MG reflects both clinical disease involvement and immune-pathological mechanisms. First, the upregulation of sphingolipids, including SM and ceramides, suggests enhanced membrane injury, inflammatory responses, and apoptosis, implying potential neuromuscular junction damage in MG. The higher SM levels observed in GMG further support its association with broader muscle involvement (Figure 3c). Second, key nodes within the glycerophospholipid metabolism pathway, such as phosphatidylethanolamine (PE) and phosphatidylinositol (PI), were markedly dysregulated. These lipids are critical for membrane stability, receptor clustering, and synaptic signaling, indicating that postsynaptic structure and neurotransmission may be impaired. Several PE species are also involved in autophagy (e.g., the ATG8–PE conjugation system), suggesting that autophagic processes may contribute to MG pathophysiology. The disruption of PE and PI was evident across both MGFA subtypes and antibody-defined subtypes (Figure 3c and Supplementary Figure S3), highlighting their dual relevance to structural damage and immune activation. Finally, the ether lipid module displayed a pronounced clinical-dimension bias. KEGG enrichment analysis revealed that the ether lipid metabolism pathway was significantly enriched only in MGFA-based comparisons, but not across antibody subtypes (Figure 3b). Consistent with this, PE(P-18:1/22:5) and PE(P-18:0/22:6) showed significant MGFA-related variation, with higher median abundances in GMG, whereas the evidence for antibody subtype-specific differences was less consistent (Figure 3c). The increased expression of ether-PE species, which are involved in membrane repair, further suggests the presence of lipid-mediated structural protection and adaptive remodeling in MG. Collectively, these findings indicate that perturbations within the ether lipid module are primarily associated with clinical disease severity, potentially reflecting broader tissue involvement and heightened neuromuscular junction stress.

### 3.4. Functional Association Between Serum Lipids and Clinical Phenotypes of MG

**Figure 4:**
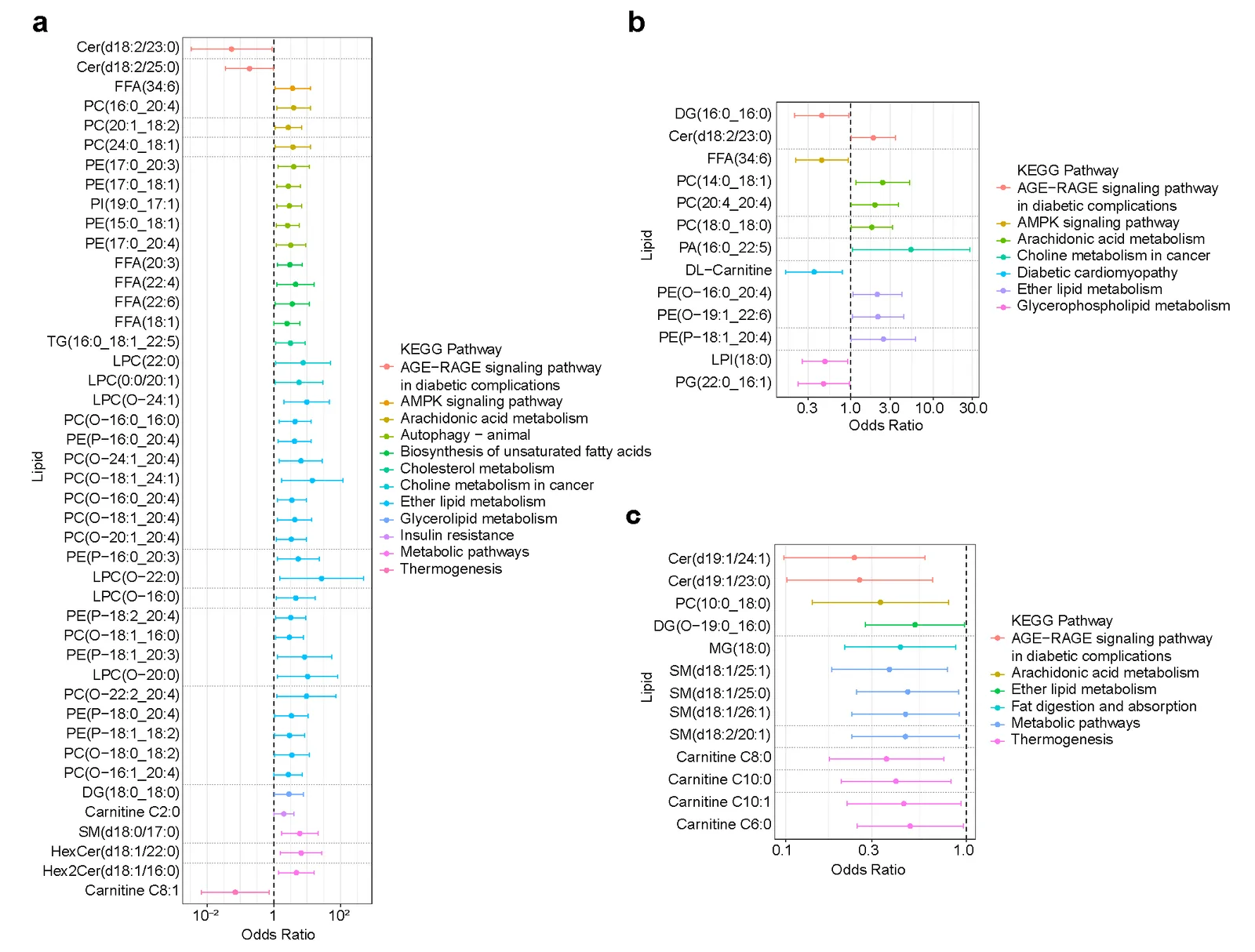
Associations between serum lipids and clinical phenotypes of MG. Forest plots summarize lipids significantly associated with disease status and MG subtypes based on multivariable logistic regression (*p* < 0.05). Circles indicate odds ratios (OR), with horizontal bars showing the 95% confidence intervals; the vertical dashed line (OR = 1) denotes the null association. Lipids are color-coded according to their major enriched KEGG pathway category. (**a**): Lipids associated with MG diagnosis compared with healthy controls (HCs). (**b**): Lipids differentiating MGFA clinical subtypes (GMG vs. OMG). (**c**): Lipids associated with antibody-defined subtypes (AChR-MG vs. dsNMG).

Given the substantial heterogeneity in clinical manifestations and therapeutic responses in MG, we further applied multivariable logistic regression to investigate the associations between circulating lipid metabolites and MG clinical phenotypes. To construct a comprehensive pool of candidate variables, all differential lipids identified across six pairwise comparisons (HCs vs. OMG, HCs vs. GMG, OMG vs. GMG, HCs vs. AChR-MG, HCs vs. dsNMG, and AChR-MG vs. dsNMG) were combined into a unified set. Each lipid was first evaluated using univariable logistic regression, followed by adjustment for demographic and clinical covariates—including age, sex, thymic and thyroid abnormalities, and immunosuppressive therapy. Lipids with adjusted *p* < 0.05 were considered significantly associated with the clinical phenotype. Forest plots (Figure 4a–c) display the odds ratio (OR) directionality and magnitude of these associations.

In total, 44 lipids were significantly associated with overall MG status, 13 were associated with MGFA clinical subtypes, and 13 were associated with antibody subtypes. Despite differences in phenotype, lipid alterations across all three dimensions converged on key metabolic pathways, including the AGE-RAGE signaling pathway, Arachidonic acid metabolism, and Ether lipid metabolism, suggesting that sphingolipid imbalance, dysregulated arachidonic acid turnover, and perturbations in ether lipid homeostasis may participate in MG-related metabolic changes.

Importantly, KEGG enrichment was consistent with the molecular identities identified in the differential analyses. For example, PC(O-24:1_20:4), PC(O-16:0_16:0), and LPC(O-24:1), which were elevated in MG relative to HCs, were also enriched in these pathways (Figure 4a). This concordance supports a potential role for ether lipids in membrane restoration or modulation of inflammatory responses. In MGFA subtype comparisons, arachidonic acid metabolism and ether lipid metabolism exhibited the highest enrichment frequency, with key nodes such as PE(P-18:1/20:4) and PE(O-16:0/20:4) corresponding to significantly elevated membrane phospholipids (Figure 4b), indicating a link between disease severity and the extent of phospholipid remodeling. Across antibody subtypes, multiple previously identified differential lipids again demonstrated significant associations (Figure 4c). The thermogenesis pathway was repeatedly enriched, consistent with the classification-dependent distribution of Carnitine C10:1 shown in the boxplots in Figure 3c. This finding may indicate differences in fatty acid metabolism among antibody-defined MG subgroups.

### 3.5. Specific Lipid Biomarker Panels for Classification of MG and Its Subtypes

We constructed diagnostic models using differential lipids from each comparison. LASSO selected top features, and the top three lipids with nonzero coefficients were used to build logistic regression classifiers. Due to sample size constraints, we used 10 × 10 repeated stratified K-fold cross-validation to estimate model generalizability.

**Figure 5:**
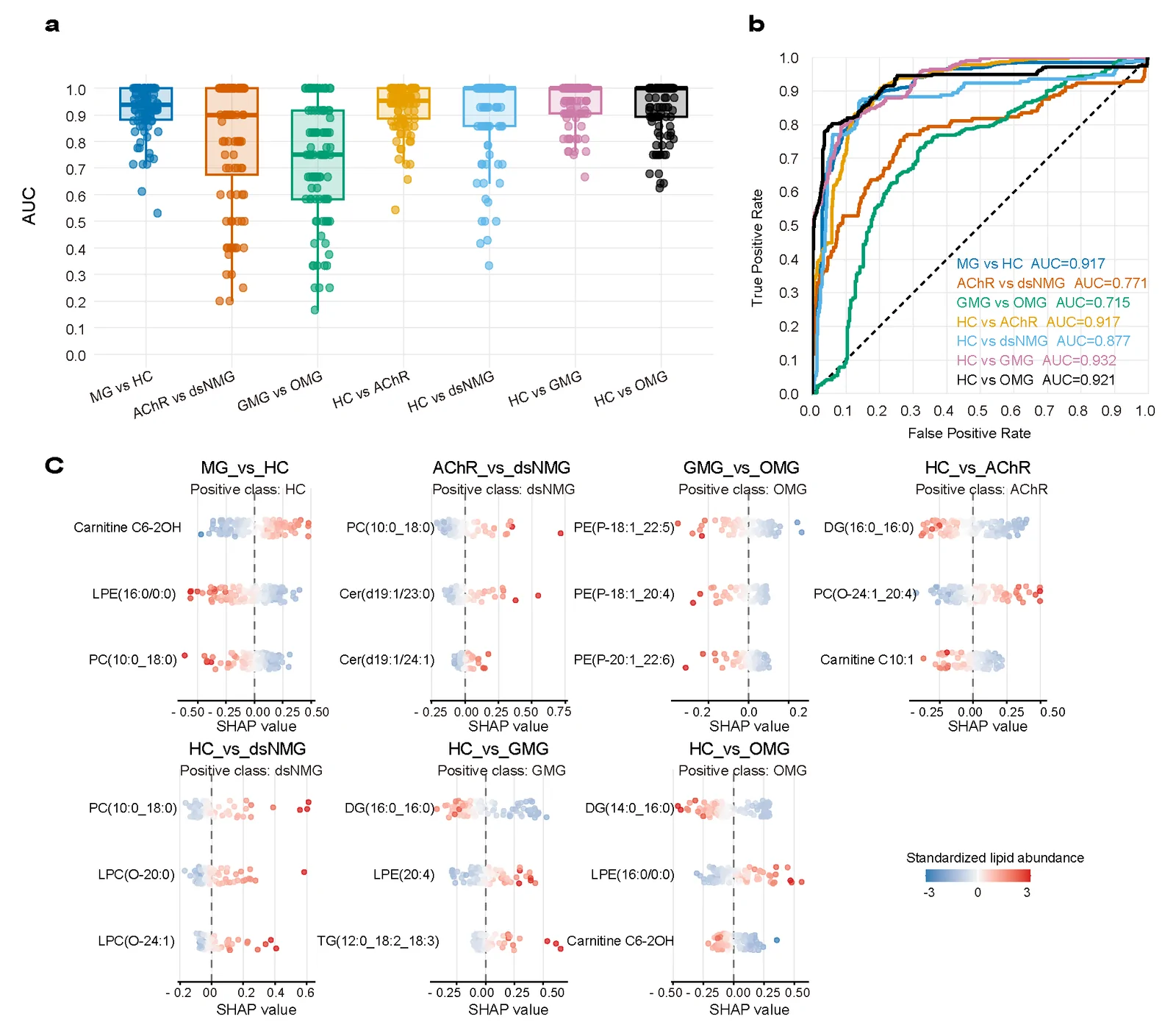
Performance and interpretability of lipid-based diagnostic models. (**a**) Distribution of classifier performance across repeated validation. Box plots summarize AUC values across 10 × 10 repeated stratified cross-validation for seven pairwise comparisons (MG vs. HCs, AChR-MG vs. dsNMG, GMG vs. OMG, HCs vs. AChR-MG, HCs vs. dsNMG, HCs vs. GMG, HCs vs. OMG). The *x*-axis indicates classification tasks, and the *y*-axis shows AUC values, illustrating the stability and robustness of each model. (**b**) Receiver operating characteristic (ROC) curves for all comparison tasks. Curves are overlaid for direct comparison of diagnostic performance. The dashed diagonal indicates random classifier performance. Corresponding AUC values with 95% confidence intervals are listed and color-coded for each model. (**c**) SHAP summary plots of the three selected lipid features for each pairwise model. Each point represents one participant, with color indicating standardized lipid abundance from low (blue) to high (red). Positive and negative SHAP values shift predictions toward the specified positive and reference classes, respectively, whereas values near zero indicate limited contribution.

As shown in Figure 5a and Supplementary Table S5, the distribution of AUC values obtained from 10-fold cross-validation demonstrated high model stability. For MG vs. HCs classification, AUC values were consistently concentrated within the 0.87–0.93 range, with tightly clustered interquartile intervals, indicating strong discrimination and minimal fluctuation across repeated training and validation cycles. Although discrimination among MG subtypes was more challenging, the models remained effective: AChR-MG vs. dsNMG yielded a mean AUC of ~0.77, and GMG vs. OMG a mean AUC of ~0.71. Importantly, when discriminating MG subtypes from HCs, the models again demonstrated high performance, with AUC distributions of 0.87–0.93 for HCs vs. AChR-MG, HCs vs. GMG, and HCs vs. OMG, suggesting that even ocular MG exhibits systemic lipid abnormalities.

Further ROC analysis (Figure 5b) confirmed the diagnostic capabilities of these models. The classifier distinguishing MG from HCs achieved an AUC of 0.917, indicating excellent discriminatory power. For subtype classification, the models yielded AUC = 0.771 for AChR-MG vs. dsNMG and AUC = 0.715 for GMG vs. OMG, demonstrating reliable identification despite subtler between-subtype lipid differences. Additionally, subtype-to-HCs classification further supported this robustness (HCs vs. AChR-MG: AUC = 0.892; HCs vs. GMG: AUC = 0.871; HCs vs. OMG: AUC = 0.921). Collectively, these findings highlight that serum lipid signatures not only distinguish MG from healthy states but also support clinically relevant subtype recognition. Compared with the conventional AChR-Ab assay, positive in only ~85% of GMG and 50–60% of OMG cases [6], the lipid-based model demonstrated superior predictive coverage and diagnostic accuracy, underscoring the potential value of lipidomics as a complementary diagnostic tool.

SHAP analysis identified the lipid features contributing most strongly to each pairwise classification model (Figure 5c). Carnitine C6-2OH, PC (10:0_18:0), and LPE (16:0/0:0) contributed prominently to MG–HC classification, while ceramides and phosphatidylethanolamines were important in the subtype models. LPC (O-24:1), LPC (O-20:0), and PC (10:0_18:0) contributed to HCs–dsNMG classification.

### 3.6. Exploratory Association Between Dynamic Changes in Differential Lipids and Clinical Improvement Based on Longitudinal Cohort

**Figure 6:**
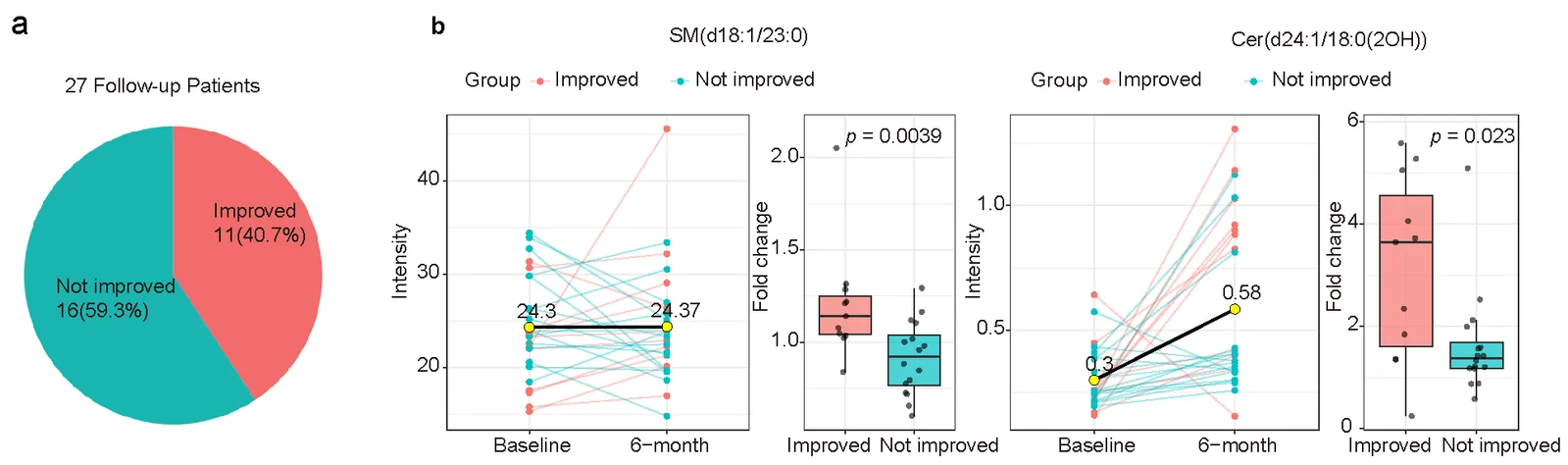
Longitudinal lipid changes associated with clinical improvement. (**a**) Twenty-seven MG patients were classified into the improved group (*n* = 11) and the not improved group (*n* = 16) based on changes in MG-ADL scores. (**b**) SM(d18:1/23:0) and Cer(d24:1/18:0(2OH)) show individual trajectories between baseline and 6-month follow-up, together with group-wise comparisons of fold changes (V6/Baseline). Each line in the trajectory plots represents an individual patient, and box plots display the median and interquartile range. Group differences were assessed using the Wilcoxon test, with *p* values indicated.

To further evaluate the association between MG-related lipidomic features and clinical improvement, longitudinal analysis was conducted in 27 patients who completed the 6-month follow-up. Baseline clinical characteristics were compared between patients who completed follow-up and those who did not (Supplementary Table S6). No statistically significant differences were observed in baseline MG-ADL score, MGFA classification, antibody subtype, thymic disease, or immunosuppressive treatment status. The detailed sample stratification is shown in Figure 6a.

Based on MG-associated lipid features identified in the cross-sectional analyses, lipid change patterns over the 6-month follow-up period were evaluated. The results showed that the sphingomyelin SM(d18:1/23:0) and the ceramide Cer(d24:1/18:0(2OH)) exhibited significantly different dynamic change patterns between patients with and without clinical improvement (Wilcoxon test, *p* < 0.05) (Figure 6b). Specifically, SM(d18:1/23:0) demonstrated a more pronounced decrease over time in patients with clinical improvement, whereas a smaller reduction was observed in patients without improvement. In contrast, Cer(d24:1/18:0(2OH)) showed a more prominent increase during follow-up in the improved group, while changes were relatively modest in the not improved group. In this exploratory analysis, SM(d18:1/23:0) and Cer(d24:1/18:0(2OH)) showed nominally significant longitudinal changes associated with clinical improvement (*p* < 0.05), warranting confirmation in larger cohorts.

Other MG-associated lipids showed similar temporal trends between groups, suggesting that longitudinal disease-related changes were mainly confined to specific sphingolipids.

## 4. Discussion

Existing research on MG has predominantly focused on proteomic and antibody-driven mechanisms [28,29,30], whereas metabolic alterations, particularly those involving lipid biology, remain largely unexplored. Here, we provide a comprehensive characterization of lipid metabolic remodeling in MG across clinical and antibody-defined subtypes. By combining serum lipidomics with classification analyses, we characterized lipid alterations across clinical and antibody-defined MG subtypes and evaluated their potential diagnostic value. These findings extend beyond the conventional paradigm of autoantibody-mediated pathology, offering new mechanistic insights and diagnostic avenues, especially for dsNMG, which lacks reliable antibody biomarkers.

Previous serum metabolomic studies established that MG is accompanied by systemic metabolic alterations, although they addressed different clinical questions. Blackmore et al. compared seropositive MG patients with healthy controls and identified a serum metabolite signature with diagnostic potential [16]. Consistent with their findings, our results demonstrated clear metabolic separation between MG and healthy states. However, our study extends this observation by resolving alterations at the lipid-species and pathway levels and by systematically comparing clinical and antibody-defined subtypes, including dsNMG. Sikorski et al. subsequently showed that baseline serum metabolomic and lipidomic profiles were associated with six-month treatment response, with changes involving glycosphingolipid and fatty acid metabolism [17]. These findings are consistent with our identification of sphingolipid and fatty-acid-related abnormalities. Nevertheless, their study primarily evaluated baseline predictors of treatment response, whereas our longitudinal analysis examined paired lipid changes during follow-up in relation to clinical improvement. Thus, the present study advances previous work through systematic subtype stratification, specific characterization of dsNMG, and longitudinal assessment of disease-associated lipid dynamics.

Among these alterations, activation of the ceramide pathway emerged as particularly prominent. Ceramides, such as Cer(d24:1/18:0(2OH)), were markedly elevated in the MG cohort, suggesting a pivotal role in disease pathophysiology. Consistent with our findings, a previous MG-specific study demonstrated that several plasma ceramides were elevated in patients with MG, correlated with QMG and MG-ADL scores, and decreased in parallel with clinical improvement [23]. Ceramide accumulation may influence membrane organization and contribute to inflammatory signaling; however, its direct effects at the NMJ cannot be determined from circulating ceramide levels alone [31]. Recent work further establishes ceramide as a important regulator of immune signaling, capable of driving chronic immune activation and tissue injury through modulation of inflammatory cascades, antigen presentation, and immune-cell adhesion and migration, underscoring its pathogenic relevance in autoimmune diseases [32]. Moreover, ceramide and S1P function as critical regulators of cell survival and death, and dysregulation of this axis has been linked to cell survival and death pathways in previous studies [33]. Its relevance to muscle-fiber regeneration and NMJ homeostasis in MG requires direct experimental investigation.

Exploratory subtype comparisons identified both shared and potentially distinct lipidomic patterns across clinical and antibody-defined MG subgroups. AChR-MG and dsNMG, despite differing antibody status, shared alterations in phospholipid and sphingolipid pathways, pointing toward core disruptions in membrane lipid architecture and signal transduction [26]. Conversely, dsNMG exhibited higher levels of several acylcarnitines and the bile-acid-related metabolite glycocholic acid, suggesting possible alterations in fatty acid and bile-acid metabolism. GMG exhibited pronounced enrichment of ether-linked PC and PE species, indicative of severe membrane remodeling and compromised membrane integrity. This aligns with GMG’s systemic severity and the recognized protective and antioxidant roles of ether phospholipids [24]. Collectively, these subtype-associated signatures provide candidate biomarkers and hypothesis-generating metabolic associations for further investigation.

KEGG pathway enrichment further connected these molecular alterations with specific biological processes. Necroptosis and sphingolipid signaling were consistently enriched across both clinical and antibody-based stratifications, highlighting consistent associations of MG with pathways related to programmed cell death and immune signaling. Pathway mapping showed that the altered sphingolipids and ceramides were enriched in pathways related to necroptosis and inflammatory signaling. Complement activation is an established mediator of NMJ injury in AChR-positive MG [34,35]. However, whether the lipid alterations identified here influence complement activation remains unknown. GP subclasses regulate membrane fluidity [36], influencing AChR clustering, synaptic transmission, and NMJ stability. Autophagy abnormalities have been reported directly in cells obtained from patients with MG, including lower autophagic activity in generalized than ocular MG; however, whether the altered PE species identified here participate in this process remains unknown [37]. Ether lipids have been proposed to contribute to membrane protection and antioxidant defense in other disease contexts [24,38], although their role in MG remains uncertain. MG-associated lipidomic alterations exhibited a dual-axis pattern associated with clinical classification and antibody-defined phenotype. Aberrations in sphingolipids and glycerophospholipids reflect the interplay between structural damage and immune stress within the neuromuscular junction microenvironment, whereas the MGFA-specific increase in ether lipid species suggests a compensatory role for these lipids in maintaining membrane homeostasis and facilitating adaptive repair during disease progression. Overall, these lipid perturbations encompass pathways related to cellular injury, autophagy, and membrane remodeling, while also delineating subtype-specific metabolic signatures across MG strata, thereby providing critical insights into the molecular underpinnings of MG. These lipid signatures may represent candidate diagnostic or disease-monitoring biomarkers, although their clinical utility requires independent validation.

Multivariable logistic regression further linked lipidomic findings with clinical relevance. Lipids associated with MG status, MGFA severity classification, and antibody subtypes were consistently enriched in the AGE-RAGE signaling pathway, arachidonic acid metabolism, and ether lipid metabolism, underscoring a central network involving arachidonic-acid-mediated inflammation and ether lipid remodeling. MGFA-specific enrichment highlighted membrane structural remodeling, whereas antibody–subtype associations prominently involved thermogenesis, consistent with differential energy metabolic burdens previously observed between GMG and dsNMG.

Using LASSO-based feature selection and repeated stratified cross-validation, we developed a lipid signature classifier that achieved excellent diagnostic performance in distinguishing MG from healthy controls (AUC = 0.917) and retained substantial capability for subtype recognition (AChR-MG vs. dsNMG: AUC = 0.77; GMG vs. OMG: AUC = 0.71). Notably, the lipid-based classifier demonstrated particular value in dsNMG (AUC = 0.877), a subgroup lacking established diagnostic biomarkers. Compared with conventional AChR-antibody assays, which show approximately 85% positivity in GMG and only 50–60% in OMG [4], lipid-based diagnostic models provided broader coverage and higher accuracy. These results support their translational potential as complementary biomarkers. SHAP analyses identified lipid features contributing most strongly to model classification, highlighting Carnitine C6-2OH, PC(10:0_18:0), and LPE(16:0/0:0) as strong predictive contributors for MG, while ether-linked LPC(O-) and PC species contributed to dsNMG classification, supporting their potential relevance as disease-associated lipid features.

In the longitudinal follow-up analysis, we further explored the association between MG-related lipid metabolic disturbances and disease status over time. Notably, SM(d18:1/23:0) and Cer(d24:1/18:0(2OH)) exhibited distinct temporal patterns in patients with and without clinical improvement, suggesting that changes in specific sphingolipid species may be associated with disease activity.

Consistent with the cross-sectional findings, Cer(d24:1/18:0(2OH)) was repeatedly identified in comparisons between patients with MG and healthy controls and among the major MG subtypes. Similarly, SM(d18:1/23:0), identified in the cross-sectional MGFA subtype analysis, showed longitudinal changes consistent with disease progression.

The directional changes in these lipids were consistent with the sphingolipid abnormalities observed in the cross-sectional analyses, supporting a potential association between sphingolipid metabolism and MG. However, given the small follow-up cohort, these preliminary associations should be regarded as hypothesis-generating rather than confirmatory. Larger longitudinal studies are needed to determine whether these lipid changes are reproducibly associated with disease activity or treatment response.

Despite the systematic characterization of lipid metabolic alterations associated with MG in this study, several limitations should be acknowledged. First, the overall cohort size was modest, and the clinical and antibody-defined subgroups were not evenly distributed. In particular, only two patients with MuSK-MG were enrolled. Given this very small sample size, MuSK-MG was included only in the descriptive characterization of the cohort and was not analyzed as an independent subgroup in inferential comparisons or predictive modeling. Moreover, although the analyses comparing AChR-MG with dsNMG and OMG with GMG included larger samples, their statistical power and generalizability remain constrained by the limited and unequal subgroup sizes. Therefore, the identified subtype-associated lipidomic signatures should be considered exploratory and require validation in larger, multi-ethnic, multicenter cohorts with more balanced representation of MG subtypes, particularly MuSK-MG. Most patients received immunosuppressive treatment, which may affect lipid metabolism. Although treatment status was adjusted for, residual confounding cannot be excluded and warrants further investigation. Finally, this observational lipidomics study identifies associations but cannot establish causal relationships between lipid alterations and MG pathophysiology. Experimental and interventional studies are required to determine whether these lipid alterations are causal contributors, compensatory responses, or secondary consequences of disease activity and treatment.

## 5. Conclusions

Serum lipidomic profiling identified 240 differential lipids between patients with MG and healthy controls. The lipid-based model distinguished MG from controls with an AUC of 0.917. Longitudinally, SM(d18:1/23:0) and Cer(d24:1/18:0(2OH)) showed distinct changes between patients with and without clinical improvement. Together, these findings characterize MG-associated lipid remodeling involving membrane homeostasis, cellular signaling, and energy metabolism across clinical and antibody-defined subtypes. These associations generate testable biological hypotheses but do not establish causal involvement in MG pathogenesis. Further validation in independent cohorts and mechanistic models is required before these lipid signatures can be translated into clinical applications.

## Figures and Tables

**Table 1:** Demographic and clinical characteristics of patients with myasthenia gravis (MG) and healthy controls (HCs).

Clinical Characteristics	MG (*n* = 68)	HCs (*n* = 68)	*p* Value
Gender, *n* (%)			
Female	43 (63.2%)	43 (63.2%)	>0.99
Male	25 (36.8%)	25 (36.8%)	
Age (years), median (IQR)	38 (20)	44 (27)	0.19
Antibody type, *n* (%)			
AChR-Ab-positive	49 (72.1%)	NA	NA
dsNMG	17 (25.0%)	NA	NA
Anti-MuSK-Ab-positive	2 (2.9%)	NA	NA
MGFA, *n* (%)			
Ocular type	37 (54.4%)	NA	NA
Generalized type	31(45.6%)	NA	NA
Diseases of thymus, *n* (%)			
YES	26 (38.2%)	NA	NA
NO	42 (61.8%)	NA	NA
Thyroid disease, *n* (%)			
YES	18 (26.5%)	NA	NA
NO	50 (73.5%)	NA	NA
Immunosuppressants, *n* (%)			
Used	56 (82.4%)	NA	NA
Not used	12 (17.6%)	NA	NA

Abbreviations: MG = myasthenia gravis; HCs = healthy controls; IQR = interquartile range; AChR = acetylcholine receptor; MuSK = muscle-specific kinase; MGFA = Myasthenia Gravis Foundation of America. Continuous variables were compared using the Mann–Whitney U test, and categorical variables were compared using the χ^2^ test or Fisher’s exact test as appropriate. NA = not applicable. All percentages were calculated based on the valid total number of cases in each group.

## Data Availability

The datasets generated and/or analysed during the current study are not publicly available due to ethical and privacy considerations but are available from the corresponding author upon reasonable request.

## Supplementary Materials

The following supporting information can be downloaded at: https://www.mdpi.com/article/10.3390/metabo16080589/s1, **Figure S1.** (a) Distribution of the 824 retained lipids among six major lipid classes. (b) Proportional composition of 43 lipid subclasses. **Figure S2.** Volcano plots for additional comparisons between MG subtypes and healthy controls. Differential lipids were defined as *p* < 0.05 and VIP > 1. **Figure S3.** Lipid-class distribution of differential features across seven pairwise comparisons. **Figure S4.** Heatmap of differential lipids (MG vs. HCs, *p* < 0.05). Rows represent lipid species and columns represent individual samples, with subgroups separated by white space. Colors reflect Z-score-normalized intensities. **Table S1.** Internal standards used for quantitative lipidomic analysis. **Table S2.** OPLS-DA model performance and permutation validation. **Table S3.** Summary of differential lipids across all comparison groups. **Table S4.** Differential lipids corresponding to Venn diagram intersections. **Table S5.** Model performance metrics and key lipid predictors for MG subgroup classification. **Table S6.** Baseline characteristics of MG patients according to 6-month follow-up status.

## Abbreviations

The following abbreviations are used in this manuscript:

AChR	Acetylcholine receptor
AChR-Ab	Acetylcholine receptor antibody
AChR-MG	Acetylcholine receptor antibody-positive myasthenia gravis
ADL	Activities of daily living
AGE-RAGE	Advanced glycation end products–receptor for advanced glycation end products
ATG8	Autophagy-related protein 8
AUC	Area under the curve
Cer	Ceramide
CI	Confidence interval
CV	Coefficient of variation
dsNMG	Double-seronegative myasthenia gravis
ELISA	Enzyme-linked immunosorbent assay
FA	Fatty acyls
FDR	False discovery rate
GL	Glycerolipids
GMG	Generalized myasthenia gravis
GP	Glycerophospholipids
GPI	Glycosylphosphatidylinositol
HCs	healthy controls
IQR	Interquartile range
KEGG	Kyoto Encyclopedia of Genes and Genomes
LASSO	Least absolute shrinkage and selection operator
LNAPE	Lyso-N-acyl phosphatidylethanolamine
LPC	Lysophosphatidylcholine
LPE	Lysophosphatidylethanolamine
MG	Myasthenia gravis
MG-ADL	Myasthenia Gravis Activities of Daily Living
MGFA	Myasthenia Gravis Foundation of America
MRM	Multiple reaction monitoring
MS/MS	Tandem mass spectrometry
MTBE	Methyl tert-butyl ether
MuSK	Muscle-specific kinase
MuSK-Ab	Muscle-specific kinase antibody
MuSK-MG	Muscle-specific kinase antibody-positive myasthenia gravis
MWDB	Metware database
NA	Not applicable
NMJ	Neuromuscular junction
OMG	Ocular myasthenia gravis
OPLS-DA	Orthogonal partial least squares–discriminant analysis
OR	Odds ratio
PA	Phosphatidic acid
PC	Phosphatidylcholine
PCA	Principal component analysis
PE	Phosphatidylethanolamine
PERMANOVA	Permutational multivariate analysis of variance
PG	Phosphatidylglycerol
PI	Phosphatidylinositol
QC	Quality control
ROC	Receiver operating characteristic
S1P	Sphingosine-1-phosphate
SHAP	SHapley Additive exPlanations
SM	Sphingomyelin
SP	Sphingolipids
TG	Triglyceride
UPLC–MS/MS	Ultra-performance liquid chromatography–tandem mass spectrometry
VIP	Variable importance in projection
