# Supplementary material for: Lipidomic Profiling Reveals Distinct Molecular Signatures Across Clinical Subtypes of Myasthenia Gravis
Source: Metabolites. 2026 Aug 19;16(8):589. doi: 10.3390/metabo16080589 (PMC13515152; doi:10.3390/metabo16080589)
Supplement: Supplementary file 1 [file metabolites-16-00589-s001.zip › STROBE_checklist.pdf]

STROBE Statement - Checklist for Case-Control Studies

Section	Item	Recommendation	Page(s) / reporting status
Title and abstract	1(a)	Indicate the study's design with a commonly used term in the title or the abstract.	p. 1
Title and abstract	1(b)	Provide in the abstract an informative and balanced summary of what was done and what was found.	pp. 1-2
Introduction - Background/rationale	2	Explain the scientific background and rationale for the investigation being reported.	pp. 2-3
Introduction - Objectives	3	State specific objectives, including any prespecified hypotheses.	pp. 2-3
Methods - Study design	4	Present key elements of study design early in the paper.	p. 3
Methods - Setting	5	Describe the setting, locations, and relevant dates, including periods of recruitment, exposure, follow-up, and data collection.	p. 3 (recruitment site and April 2024-April 2025; 6-month follow-up)
Methods - Participants	6(a)	Give the eligibility criteria, and the sources and methods of case ascertainment and control selection. Give the rationale for the choice of cases and controls.	p. 3
Methods - Participants	6(b)	For matched studies, give matching criteria and the number of controls per case.	pp. 3, 7-9 (age- and sex-matched controls; 68 cases and 68 controls, 1:1). The exact age-matching tolerance is not reported.
Methods - Variables	7	Clearly define all outcomes, exposures, predictors, potential confounders, and effect modifiers. Give diagnostic criteria, if applicable.	pp. 3, 5-7
Methods - Data sources/measurement	8*	For each variable of interest, give sources of data and details of methods of assessment (measurement). Describe comparability of assessment methods if there is more than one group.	pp. 3-7
Methods - Bias	9	Describe any efforts to address potential sources of bias.	pp. 3, 5-7 (age/sex matching, standardized sampling and QC, transformation/scaling, covariate adjustment, permutation testing, and repeated cross-validation)
Methods - Study size	10	Explain how the study size was arrived at.	NA
Methods - Quantitative variables	11	Explain how quantitative variables were handled in the analyses. If applicable, describe which groupings were chosen and why.	pp. 5-7
Methods - Statistical methods	12(a)	Describe all statistical methods, including those used to control for confounding.	pp. 5-7
Methods - Statistical methods	12(b)	Describe any methods used to examine subgroups and interactions.	pp. 5-7
Methods - Statistical	12(c)	Explain how missing data were addressed.	pp. 5-6 (row-median and median imputation)

Section	Item	Recommendation	Page(s) / reporting status
methods			
Methods - Statistical methods	12(d)	If applicable, explain how matching of cases and controls was addressed.	pp. 3, 6-7 (age/sex matching reported; regression models included age and sex as covariates). A dedicated matched-analysis method is not reported.
Methods - Statistical methods	12(e)	Describe any sensitivity analyses.	No formal sensitivity analysis reported. Model robustness was assessed using permutation testing and repeated stratified cross-validation (pp. 5-7).
Results - Participants	13(a)	Report numbers of individuals at each stage of study, including eligibility, inclusion, follow-up, and analysis.	pp. 7-8, 17
Results - Participants	13(b)	Give reasons for non-participation at each stage.	NA
Results - Participants	13(c)	Consider use of a flow diagram.	pp. 7-8 (Figure 1); p. 17 (Figure 6 for longitudinal follow-up)
Results - Descriptive data	14(a)	Give characteristics of study participants and information on exposures and potential confounders.	pp. 8-9 (Table 1); p. 17 and Supplementary Table 6 for the follow-up subset
Results - Descriptive data	14(b)	Indicate the number of participants with missing data for each variable of interest.	NA
Results - Outcome data	15*	Report numbers in each exposure category, or summary measures of exposure.	pp. 7-9 (group sizes and participant characteristics); pp. 9-15 (summary lipidomic findings)
Results - Main results	16(a)	Give unadjusted estimates and, if applicable, confounder-adjusted estimates and their precision. Make clear which confounders were adjusted for and why.	pp. 6, 9-15; adjusted odds ratios with 95% CIs are presented on pp. 14-15
Results - Main results	16(b)	Report category boundaries when continuous variables were categorized.	p. 3 (clinical-improvement threshold: reduction in MG-ADL score of at least 2 points); otherwise not applicable to continuous lipid intensities
Results - Main results	16(c)	If relevant, consider translating estimates of relative risk into absolute risk for a meaningful time period.	Not applicable; the study did not estimate relative risks.
Results - Other analyses	17	Report other analyses performed, such as subgroup, interaction, and sensitivity analyses.	pp. 9-17 (clinical- and antibody-defined subgroup, pathway, prediction-model, and longitudinal analyses)
Discussion - Key results	18	Summarise key results with reference to study objectives.	pp. 18-20
Discussion - Limitations	19	Discuss limitations, taking into account sources of potential bias or imprecision, including direction and magnitude where possible.	p. 20
Discussion - Interpretation	20	Give a cautious overall interpretation considering objectives, limitations, multiplicity of analyses, similar studies, and other relevant evidence.	pp. 18-20
Discussion - Generalisability	21	Discuss the generalisability (external validity) of the study results.	p. 20
Other information - Funding	22	Give the source of funding and the role of the funders for the present study and, if applicable, for the original study on which it is based.	p. 21 (funding sources reported; funders' role not explicitly stated)

Note: An Explanation and Elaboration article discusses each checklist item and gives methodological background and published examples of transparent reporting. The STROBE checklist is best used in conjunction with this article (freely available on the Web sites of PLoS Medicine at http://www.plosmedicine.org/, Annals of Internal Medicine at http://www.annals.org/, and Epidemiology at http://www.epidem.com/). Information on the STROBE Initiative is available at http://www.strobe-statement.org.
