# Supplementary material for: Lipidomic Profiling Reveals Distinct Molecular Signatures Across Clinical Subtypes of Myasthenia Gravis
Source: Metabolites. 2026 Aug 19;16(8):589. doi: 10.3390/metabo16080589 (PMC13515152; doi:10.3390/metabo16080589)
Supplement: Supplementary file 1 [file metabolites-16-00589-s001.zip › Supplementary Figure.pdf]

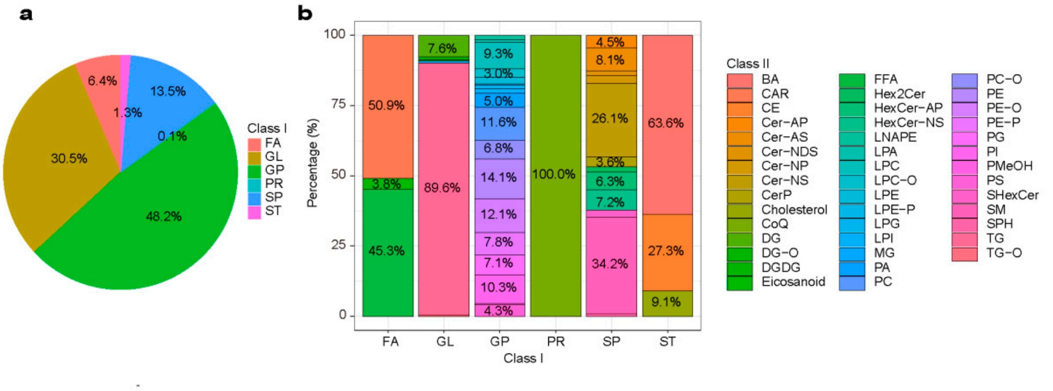

Supplementary Figure S1: (a) Distribution of the 824 retained lipids among six major lipid classes. (b) Proportional composition of 43 lipid subclasses.

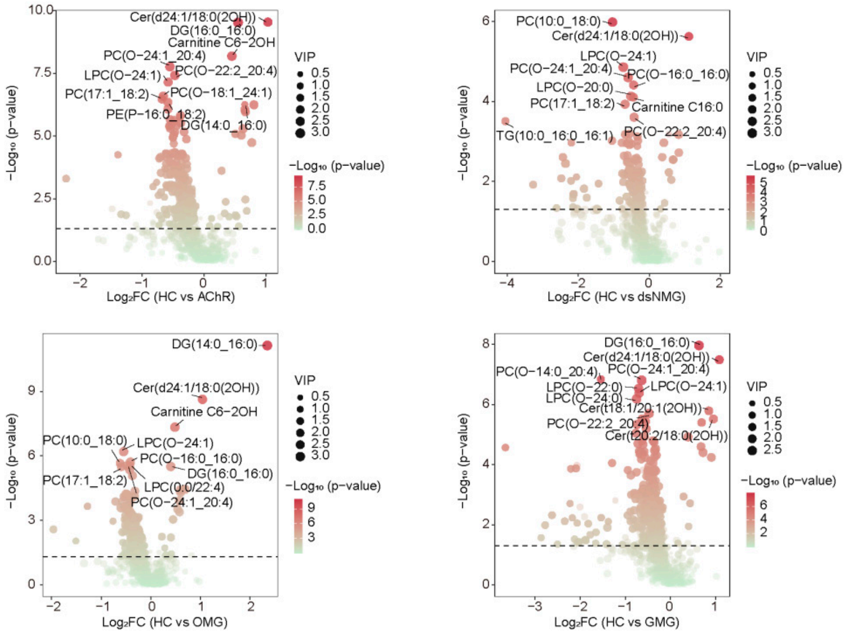

Supplementary Figure S2. Volcano plots for additional comparisons between MG subtypes and healthy controls; differential lipids were defined as P < 0.05 and VIP > 1.

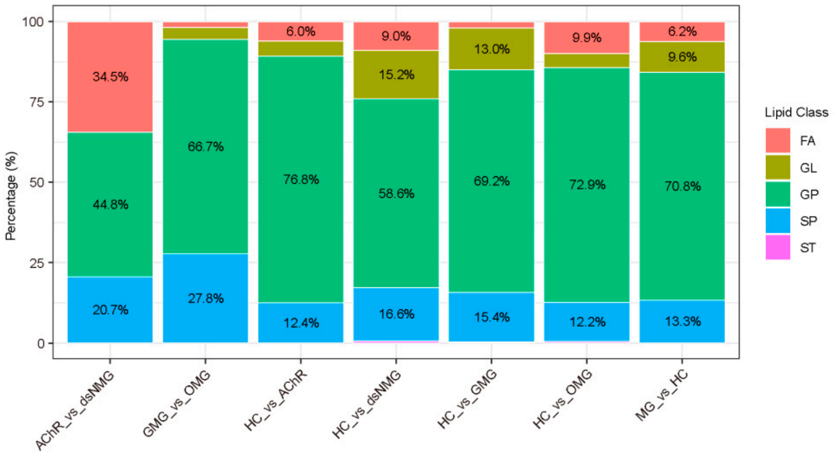

Supplementary Figure S3: Lipid-class distribution of differential features across seven pairwise comparisons.

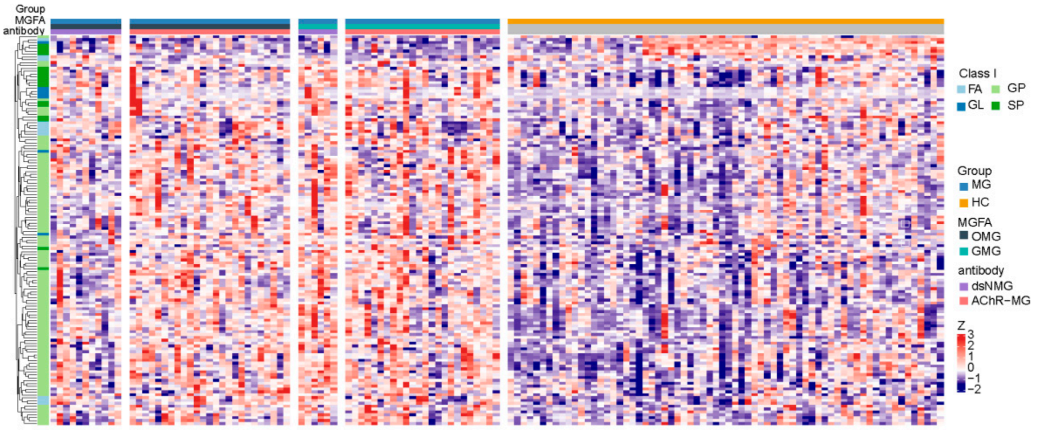

Supplementary Figure S4: Heatmap of differential lipids (MG vs HCs, P < 0.05). Rows represent lipid species and columns represent individual samples, with subgroups separated by white space. Colors reflect Z-score-normalized intensities.
