# Supplementary material for: Lipidomic Profiling Reveals Distinct Molecular Signatures Across Clinical Subtypes of Myasthenia Gravis
Source: Metabolites. 2026 Aug 19;16(8):589. doi: 10.3390/metabo16080589 (PMC13515152; doi:10.3390/metabo16080589)
Supplement: Supplementary file 1 [file metabolites-16-00589-s001.zip › Supplementary_Table_S1.pdf]

Supplementary Table S1. Internal standards used for quantitative lipidomic analysis

No.	Lipid class	Abbreviation	Internal standard	CAS No.	Manufacturer	Catalog No.	Concentration (nmol/mL)
1	Phosphatidylcholine	PC	PC(16:0/16:0)-d9	77165-56-1	Avanti Polar Lipids	860352P	0.2
2	Eicosanoid	Eicosanoid	5S-HETE-d8	330796-62-8	Cayman Chemical	334230	0.04
3	Free fatty acid	FFA	Arachidonic Acid-d8	69254-37-1	Cayman Chemical	390010	0.2
4	Acylcarnitine	CAR	CAR(16:0)-d3	202480-73-7	Sigma-Aldrich	55107	0.04
5	Cholesteryl ester	CE	CE(18:1)-d7	1416275-35-8	Avanti Polar Lipids	700185M	2
6	Ceramide	Cer	Cer(d18:1/15:0)-d7	—	Avanti Polar Lipids	860681P	0.1
7	Ceramide	Cer	Cer(d18:1-d7/16:0)	—	Avanti Polar Lipids	860676P	0.1
8	Ceramide	Cer	Cer(d18:1-d7/18:0)	—	Avanti Polar Lipids	860677P	0.1
9	Ceramide	Cer	Cer(d18:1-d7/24:0)	1840942-15-5	Avanti Polar Lipids	860678P	0.1
10	Ceramide	Cer	Cer(d18:1-d7/24:1)	54164-50-0	Avanti Polar Lipids	860525P	0.1
11	Phytoceramide	Cert	Cer(t18:0/22:0-d3)	2011762-87-9	Cayman Chemical	9003465	0.1
12	Ceramide 1-phosphate	CerP	CerP(d18:1/8:0)	474943-70-9	Avanti Polar Lipids	860532P	0.4
13	Cholesterol	Cho	cholesterol-d7	83199-47-7	Sigma-Aldrich	700041P	5
14	Coenzyme Q	CoQ	CoQ10-d9	303-98-0	Avanti Polar Lipids	802891	0.2
15	Diacylglycerol	DG	DG(17:0/17:0)-d5	—	Avanti Polar Lipids	800854P	0.2
16	Free fatty acid	FFA	FFA(16:0)-d31	39756-30-4	Supelco	68277	0.2
17	Bile acid	BA	GCDCA-d4	1201918-16-2	Sigma-Aldrich	739715	0.2
18	Hexosylceramide	HexCer	HexCer(d18:1-d5/18:0)	2260670-13-9	Avanti Polar Lipids	860638P	0.4
19	Lysophosphatidic acid	LPA	LPA(17:0)	799268-66-9	Avanti Polar Lipids	857127P	0.5
20	Lysophosphatidylcholine	LPC	LPC(15:0)-d5	2342574-95-0	Avanti Polar Lipids	870309L	0.2
21	Lysophosphatidylcholine	LPC	LPC(16:0)-d31	327178-91-6	Avanti Polar Lipids	860397P	0.2
22	Lysophosphatidylcholine	LPC	LPC(17:0)-d5	2342575-12-4	Avanti Polar Lipids	855679L	0.2
23	Lysophosphatidylcholine	LPC	LPC(18:1)-d7	2097561-13-0	ZZStandard	IR-54029	0.2
24	Lysophosphatidylethanolamine	LPE	LPE(15:0)-d5	2342574-96-1	Avanti Polar Lipids	856709L	0.2
25	Lysophosphatidylethanolamine	LPE	LPE(18:1)-d7	2260669-47-2	ZZStandard	IR-54035	0.2
26	Lysophosphatidylglycerol	LPG	LPG(15:0)-d5	2342574-98-3	ZZStandard	IR-54057	0.2
27	Lysophosphatidylglycerol	LPG	LPG(17:1)	1246298-11-2	ZZStandard	ZL-20103	0.2

No.	Lipid class	Abbreviation	Internal standard	CAS No.	Manufacturer	Catalog No.	Concentration (nmol/mL)
28	Lysophosphatidylinositol	LPI	LPI(17:1)	1246353-39-8	Avanti Polar Lipids	850103P	0.2
29	Lysophosphatidylserine	LPS	LPS(17:1)	1246298-15-6	Avanti Polar Lipids	858141P	0.2
30	Monoacylglycerol	MG	MG(18:1)-d7	2260669-49-4	Avanti Polar Lipids	791646C	0.5
31	Phosphatidic acid	PA	PA(17:0/17:0)	154804-54-3	Avanti Polar Lipids	830856P	0.5
32	Phosphatidylcholine	PC	PC(15:0/18:1(d7))	2097561-16-3	ZZStandard	IR-54021	0.2
33	Phosphatidylcholine	PC	PC(16:0-d31/18:1)	179093-76-6	Avanti Polar Lipids	860399P	0.2
34	Phosphatidylcholine	PC	PC14:0/14:0-d9	71479-88-4	Avanti Polar Lipids	860342P	0.2
35	Phosphatidylethanolamine	PE	PE(15:0/18:1(d7))	2097561-15-2	ZZStandard	IR-54033	0.2
36	Phosphatidylethanolamine	PE	PE(16:0-d31/18:1)	326495-44-7	Avanti Polar Lipids	860374P	0.2
37	Phosphatidylethanolamine	PE	PE(17:0/14:1)-d5	2342575-78-2	Avanti Polar Lipids	856721L	0.2
38	Phosphatidylethanolamine	PE	PE(17:0/18:1)-d5	2342575-56-6	Avanti Polar Lipids	856719L	0.2
39	Phosphatidylethanolamine	PE	PE(17:0-22:4)-d5	2342575-44-2	ZZStandard	IR-54054	0.2
40	Phosphatidylglycerol	PG	PG(15:0/18:1(d7))	—	Avanti Polar Lipids	791640C	0.4
41	Phosphatidylglycerol	PG	PG(16:0/d31/18:1)	327178-87-0	Avanti Polar Lipids	860384P	0.4
42	Phosphatidylinositol	PI	PI(16:0-d31/18:1)	799812-61-6	Avanti Polar Lipids	860042P	0.2
43	Phosphatidylmethanol	PMeOH	PMeOH(16:0/16:0)	92609-89-7	ZZStandard	ZL-20797	0.1
44	Phosphatidylserine	PS	PS(15:0/18:1(d7))	—	Avanti Polar Lipids	791639C	0.4
45	Phosphatidylserine	PS	PS(16:0(d31)/18:1)	327178-96-1	Avanti Polar Lipids	860403P	0.4
46	Phosphatidylserine	PS	PS(16:0/16:0)-d9	28152	Cayman Chemical	28152	0.4
47	Sphingomyelin	SM	SM(d18:1/18:1)-d9	2260669-50-7	Avanti Polar Lipids	791649C	0.2
48	Sphingomyelin	SM	SM(d18:1/20:1)-d9	2342574-48-3	Avanti Polar Lipids	860742L	0.2
49	Sphingomyelin	SM	SM(d18:1/22:1)-d9	2342574-51-8	Avanti Polar Lipids	860743L	0.2
50	Sphingomyelin	SM	SM(d18:1-d9/15:0)	—	Avanti Polar Lipids	860686P	0.2
51	Sphingosine	SPH	SPH(18:1)-d7	1246304-34-6	Avanti Polar Lipids	860657P	0.04
52	Triacylglycerol	TG	TG(14:0/16:1/14:0)-d5	944709-23-3	ZZStandard	IR-54066	0.2
53	Triacylglycerol	TG	TG(16:0-15:1-16:0)-d5	2342574-85-8	Avanti Polar Lipids	860910L	0.2
54	Triacylglycerol	TG	TG(17:0/17:1/17:0)-d5	958760-74-2	Avanti Polar Lipids	860903P	0.2
