# Supplementary material for: Lipidomic Profiling Reveals Distinct Molecular Signatures Across Clinical Subtypes of Myasthenia Gravis
Source: Metabolites. 2026 Aug 19;16(8):589. doi: 10.3390/metabo16080589 (PMC13515152; doi:10.3390/metabo16080589)
Supplement: Supplementary file 1 [file metabolites-16-00589-s001.zip › Supplementary_Table_S2.pdf]

Supplementary Table S2. OPLS-DA model performance and permutation validation

Comparison	R^2X (cum)	R^2Y (cum)	Q^2 (cum)	Q^2 permutation P	Q^2 exceedances	R^2Y permutation P	R^2Y exceedances
MG vs HCs	0.378	0.869	0.622	<0.005	0/200	<0.005	0/200
HCs vs OMG	0.352	0.831	0.456	<0.005	0/200	<0.005	0/200
HCs vs GMG	0.374	0.860	0.555	<0.005	0/200	<0.005	0/200
HCs vs AChR-MG	0.360	0.840	0.578	<0.005	0/200	<0.005	0/200
HCs vs dsNMG	0.442	0.939	0.455	<0.005	0/200	<0.005	0/200

Note. R^2X indicates the proportion of variance in X explained by the model; R^2Y indicates goodness of fit for class membership; Q^2 indicates cross-validated predictive ability. Permutation P values were calculated from 200 class-label permutations. $P < 0.005$ corresponds to 0 of 200 permuted models equaling or exceeding the observed statistic. Models were classified as validated when both R^2Y and Q^2 permutation tests were significant and Q^2 was positive.
