# Supplementary material for: Lipidomic Profiling Reveals Distinct Molecular Signatures Across Clinical Subtypes of Myasthenia Gravis
Source: Metabolites. 2026 Aug 19;16(8):589. doi: 10.3390/metabo16080589 (PMC13515152; doi:10.3390/metabo16080589)
Supplement: Supplementary file 1 [file metabolites-16-00589-s001.zip › supplementary_Table_S6.pdf]

Supplementary Table S6. Baseline characteristics of MG patients according to 6-month follow-up status

Characteristic	Overall MG cohort (n=68)	Completed follow-up (n=27)	Did not complete follow-up (n=41)	P value
MG-ADL, median (IQR)	3 (2–5)	3 (2–6)	3 (2–5)	.657
**MGFA, n (%)**				**.400**
Ocular type	37 (54.4%)	13 (48.1%)	24 (58.5%)	
Generalized type	31 (45.6%)	14 (51.9%)	17 (41.5%)	
**Antibody type, n (%)**				**.371**
AChR-Ab-positive	49 (72.1%)	22 (81.5%)	27 (65.9%)	
dsNMG	17 (25.0%)	5 (18.5%)	12 (29.3%)	
Anti-MuSK-Ab-positive	2 (2.9%)	0 (0.0%)	2 (4.9%)	
**Diseases of thymus, n (%)**				**.869**
Yes	26 (38.2%)	10 (37.0%)	16 (39.0%)	
No	42 (61.8%)	17 (63.0%)	25 (61.0%)	
**Thyroid disease, n (%)**				**.020**
Yes	18 (26.5%)	3 (11.1%)	15 (36.6%)	
No	50 (73.5%)	24 (88.9%)	26 (63.4%)	
**Immunosuppressants, n (%)**				**.106**
Use	56 (82.4%)	25 (92.6%)	31 (75.6%)	
Not use	12 (17.6%)	2 (7.4%)	10 (24.4%)	

Data are presented as median (IQR) or n (%).
P values represent comparisons between patients who completed the 6-month follow-up and those who did not; the overall MG cohort is shown descriptively.
MG-ADL was compared using the Mann–Whitney U test. Categorical variables were compared using Pearson's chi-square test or Fisher's exact test, as appropriate.
All clinical characteristics and treatment variables refer to baseline status.
Abbreviations: MG, myasthenia gravis; IQR, interquartile range; MG-ADL, Myasthenia Gravis Activities of Daily Living; MGFA, Myasthenia Gravis Foundation of America; AChR-Ab, acetylcholine receptor antibody; MuSK-Ab, muscle-specific kinase antibody; dsNMG, double-seronegative myasthenia gravis.
